# Waveform-Prediction Augmentation and Deep Manifold Learning Enable Imbalanced Fault Diagnosis in Rotating Machinery

**DOI:** 10.3390/s26175581

**Published:** 2026-09-02

**Authors:** Yu Tian, Shunsheng Guo, Yibing Li, Xin Zhang, Li Jiang

**Affiliations:** 1 School of Mechanical and Electronic Engineering, Wuhan University of Technology, Wuhan 430070, China; 279048@whut.edu.cn (Y.T.); guoshunsheng@whut.edu.cn (S.G.); ahlyb@whut.edu.cn (Y.L.); 2 Hubei Digital Manufacturing Key Laboratory, Wuhan University of Technology, Wuhan 430070, China; 3 Mechanical and Aerospace Engineering, The Hong Kong University of Science and Technology, Hong Kong SAR, China; mexzyl@ust.hk

**Keywords:** rotating machinery, fault diagnosis, imbalanced data, nonlinear autoregressive neural network, dynamic time warping, manifold learning

## Abstract

Fault diagnosis of rotating machinery is essential for ensuring the reliable and safe operation of industrial equipment. However, imbalanced training data often bias intelligent diagnostic models toward majority classes, resulting in insufficient representation of minority faults. In addition, the high dimensionality and redundancy of vibration signals make it difficult to extract discriminative features and preserve meaningful neighborhood structures, thereby degrading diagnostic performance. To address these challenges, a waveform-prediction augmentation and deep manifold learning model for imbalanced fault diagnosis is proposed for rotating machinery. First, minority-class fault signals are augmented through time-series prediction using a nonlinear autoregressive neural network (NARNN), thereby alleviating the class imbalance at the data level. Subsequently, a deep manifold feature mapping (DMAP) is proposed to extract discriminative fault features. In DMAP, a stacked autoencoder (SAE) is employed to perform preliminary feature extraction on the training samples and obtain deep latent feature representations. The deep features extracted by the SAE are further processed using a dynamic time warping (DTW)-assisted manifold learning method for dimensionality reduction. In this process, DTW is used to calculate pairwise dissimilarities between deep representations and construct the neighborhood graph, thereby better preserving the local neighborhood relationships and topological structure of the deep features and producing more discriminative low-dimensional representations. Finally, a *k*-nearest neighbor classifier is adopted for fault classification. By integrating data augmentation, deep feature learning, and manifold dimensionality reduction, the proposed model effectively improves the fault diagnosis performance in imbalanced scenarios. Experiments on the bearing and planetary gearbox datasets demonstrate that, under an imbalance ratio of 0.1, the proposed method improves diagnostic accuracy by 10.2 and 1.6 percentage points, respectively. The comparative results further confirm its effectiveness in alleviating the diagnostic difficulties caused by class imbalance.

## 1. Introduction

Rotating machinery is a fundamental component of modern industry and has been extensively applied in manufacturing, transportation, aerospace engineering, and wind power systems. Bearings and gears are key elements in rotating machinery. Their degradation or failure directly affects the operational stability and reliability of the entire system and may cause severe economic losses or accidents. Therefore, condition monitoring and fault diagnosis of rotating machinery are of great practical significance. In recent years, with the rapid development of machine learning and deep learning techniques, intelligent fault diagnosis (IFD) has attracted increasing attention and has gradually become an active research topic [1,2,3]. However, traditional IFD methods require sufficient and well-balanced training data to achieve satisfactory performance. In most cases, fault samples are usually much scarcer than normal samples due to the low occurrence probability of faults and the high cost of destructive experiments. This imbalance limits the ability of existing methods to adequately characterize minority fault classes, causing the classifier to be biased toward majority classes and degrading the diagnostic performance for minority samples in practical scenarios [4]. Moreover, the insufficient extraction of fault-discriminative information further weakens the generalization ability of the model [5]. Therefore, improving the diagnostic ability of intelligent models in imbalanced scenarios remains a critical challenge in rotating machinery fault diagnosis.

To alleviate the adverse influence of class imbalance, various imbalanced fault diagnosis methods have been developed. These approaches are commonly divided into three categories: data-level, feature-level, and classifier-level methods. Feature-level methods improve the feature representation of minority fault classes through feature optimization, ensemble learning [6], and transfer learning [7]. Classifier-level methods, such as cost-sensitive learning [8] and boundary modulation [9], improve imbalanced diagnosis by modifying the training objective or optimization strategy of the classifier. As the most direct strategy, data-level methods aim to modify the composition of the training set to alleviate the imbalance. Cao et al. used multi-step downsampling to capture multi-scale temporal representations from raw vibration signals to improve small-sample fault diagnosis performance [10]. Dong et al. improved minority sample representation through density-aware oversampling [11]. Ma et al. proposed an abductive reasoning and physical model guided multi-scale synthetic minority oversampling technique (ARPMG-MS-SMOTE) framework that uses multi-scale synthetic minority oversampling technique (SMOTE) to synthesize structurally coherent minority bearing fault samples from physics-informed latent features, thereby improving fault diagnosis under extreme class imbalance and limited faulty data [12]. Wang et al. proposed an model-data-driven sample generation (MDDSG) framework that uses a threshold-guided generative adversarial network (GAN) to generate high-quality synthetic bearing fault samples, thereby alleviating data imbalance and improving diagnostic robustness under scarce fault data [13]. However, downsampling may discard useful fault information, while many existing sample-level data expansion methods increase the risk of overfitting and are often insufficient to characterize the nonlinear dynamic evolution of vibration signals. Moreover, the temporal dependencies and dynamic evolution patterns inherent in fault vibration signals are not fully considered. To address this issue, data augmentation based on time series prediction provides a feasible way to extend minority vibration signals while preserving their temporal continuity. Previous studies have shown that nonlinear autoregressive neural networks (NARNNs) has strong predictive capability for nonlinear and non-stationary periodic signals [14]. Wang et al. used an NARNN to predict future degradation trends from the extracted vibration-signal time-series features [15]. Gong et al. applied an NARNN to predict future trends of aging features from historical data, thereby providing extended feature inputs for accurate battery aging trajectory prediction [16]. Gidom et al. investigated an optimized NARNN model for short-term wind speed forecasting, demonstrating its strong capability in predicting stochastic wind-speed time-series data with competitive accuracy and lower computational cost [17]. More directly, Zhou et al. proposed an imbalanced fault diagnosis method where NARNN is successfully used to predict and expand minority fault samples, thereby balancing different health-condition datasets before continuous wavelet transform (CWT)-based time–frequency transformation and convolutional neural network (CNN) classification [14]. Therefore, by predicting the subsequent evolution of minority fault signals, NARNNs can generate additional samples with temporal continuity, thereby alleviating the imbalance problem while retaining time-series-related fault information. However, the augmented high-dimensional vibration samples still contain redundant and nonlinear information. Therefore, extracting compact and discriminative fault features from these samples remains a further challenge.

Conventional dimensionality reduction methods, including principal component analysis (PCA) [18] and linear discriminant analysis (LDA) [19], have been commonly adopted for feature representations. PCA projects high-dimensional data onto directions with maximum variance, while LDA aims to find a discriminative subspace that improves class separability. However, these methods are essentially linear and may be insufficient to describe the complex nonlinear structures embedded in vibration signals. To capture the underlying geometry of complex high-dimensional data, manifold learning methods have been increasingly applied to fault feature extraction, such as local linear embedding (LLE) [20], Laplacian eigenmaps (LE) [21], locality preserving projection (LPP) [22], Isometric feature mapping (Isomap) [23], and local tangent space alignment (LTSA) [24]. Among these methods, uniform manifold approximation and projection (UMAP), proposed by McInnes et al. [25], has received considerable attention because it can preserve local neighborhood information while retaining part of the global data structure. Specifically, UMAP builds a fuzzy topological graph in the original feature space and learns a compact low-dimensional embedding through optimization, showing favorable performance in visualization and dimensionality reduction tasks. On this basis, McConville et al. developed the (Not Too) Deep Clustering framework (N2D), in which an autoencoder is first used to learn latent representations, followed by UMAP to uncover the underlying manifold [26]. This demonstrates the advantage of combining deep feature learning with nonlinear manifold representation. Chen et al. introduced adaptive weighted distance manifold learning (AWDU) into bogie bearing fault diagnosis, where adaptive weighted distance and adjacency relationships were used to guide the low-dimensional embedding of high-dimensional vibration features [27].

However, most existing manifold learning methods rely on Euclidean distance to construct neighborhood graphs, which may not adequately capture the nonlinear structure of vibration-signal features and may therefore lead to inaccurate neighborhood connections. Dynamic time warping (DTW) [28] provides a flexible dissimilarity measure by capturing nonlinear correspondences between signal representations and has been widely applied to fault diagnosis. Gao et al. proposed dynamic time warping Gaussian spatiotemporal self-attention network (DGSSANet) for fault diagnosis in intermittent industrial processes, where DTW is used to transform unequal-length sensor signals into equal-dimensional warping matrices and enhance the modeling of temporal similarity [29]. Sun et al. introduced a two-stage DTW-based fault detection method for rotating machinery under variable speeds, where a fixed-speed reference signal is estimated to guide DTW in determining optimal warping paths for signal regularization, and order-domain DTW distances are further used for fault type identification [30]. Li et al. proposed semisupervised high-order graph diffusion network (SHGDN) for cross-domain rotating machinery fault diagnosis, in which FastDTW is used to measure the similarity of discriminative subsequences called Shapelets and construct a dynamic graph with semantic perception ability [31]. Therefore, DTW-derived similarity can better characterize nonlinear relationships between signal representations and support the construction of a more reliable neighborhood graph, thereby facilitating the preservation of local topological structure during low-dimensional mapping.

Based on the above analysis, two limitations can be identified in existing imbalanced fault diagnosis studies:

(1) Most data augmentation methods mainly improve class distribution by increasing the number of minority class samples, but they pay insufficient attention to the temporal evolution characteristics of fault signals.
(2) The high dimensionality and redundancy of vibration signals pose challenges to discriminative feature extraction and neighborhood construction. Moreover, different dissimilarity functions affecting neighborhood construction are insufficiently considered.

Aimed at the two key issues, this paper proposes a waveform-prediction augmentation and deep manifold learning for imbalanced fault diagnosis in rotating machinery. An NARNN is first used to augment minority-class fault signals. An SAE then extracts compact latent representations from the augmented samples. Subsequently, DTW is incorporated into UMAP to achieve similarity measurement during manifold construction. The main contributions of this paper are summarized as follows.

(1) An NARNN-based signal augmentation strategy is developed for minority fault classes. By exploiting time-series prediction, this strategy expands minority-class samples and reduces class imbalance, while retaining the temporal dynamic information of vibration signals.
(2) A deep manifold feature mapping (DMAP) method is developed by combining an SAE and DTW-enhanced UMAP. The SAE is used to extract compact latent features, while DTW is introduced as a dissimilarity function to construct a neighborhood graph during manifold construction.
(3) An integrated imbalanced fault diagnosis framework is established by combining data-level augmentation, deep feature learning, manifold dimensionality reduction, and KNN classification. The framework improves the representation and recognition ability of minority fault categories.
(4) Experiments are conducted on bearing and gearbox datasets under different imbalance ratios. The results indicate that the proposed method achieves better diagnostic performance than several representative methods, demonstrating its effectiveness for imbalanced rotating machinery fault diagnosis.

The remainder of this paper is structured as follows. Section 2 introduces the principles of the NARNN, SAE, and UMAP. Section 3 describes the proposed fault diagnosis framework in detail. Section 4 reports the experimental validation on bearing and gearbox datasets. Section 5 concludes the paper and discusses future work.

## 2. Theoretical Basis

### 2.1. Nonlinear Autoregression Neural Network

A nonlinear autoregression neural network (NARNN) is a nonlinear extension of the traditional autoregressive model. It replaces the linear mapping in the autoregressive model with a nonlinear function approximated by a feedforward neural network. Therefore, the NARNN has been widely used for time-series forecasting tasks involving complex dynamics. For a univariate time series, NARNN predicts the current output based on several previous outputs. Its general form can be written as:

(1) x(t)=f(x(t−1),x(t−2),x(t−3)…x(t−n))

where *x*(*t*) represents the output at time *t*, *f*(·) denotes the nonlinear mapping function learned by the neural network, and *n* is the delay order. Therefore, the current output is related to the outputs of the preceding *n* time steps.

**Figure 1:**
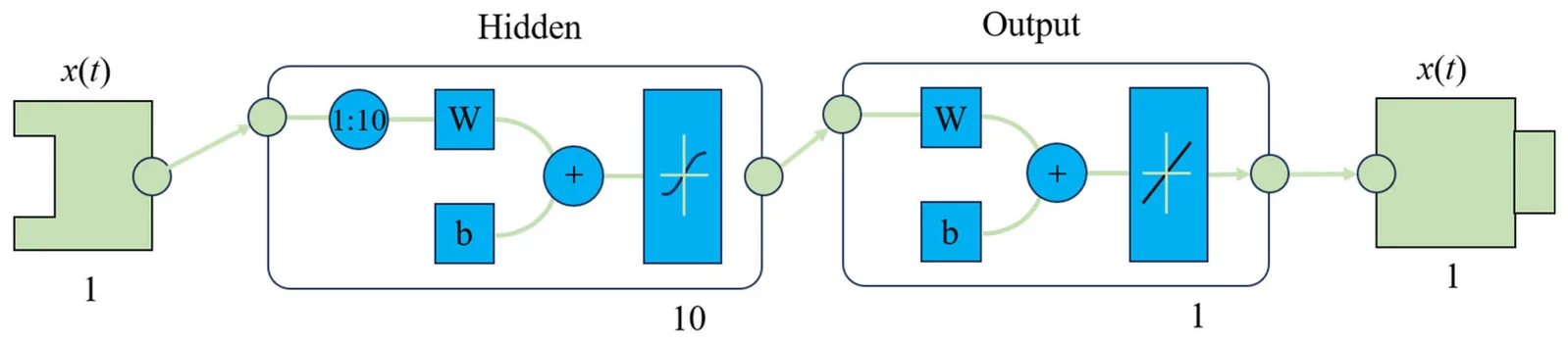
The topological architecture of the NARNN.

Figure 1 shows the basic topology of the NARNN model. The network mainly consists of an input layer, hidden layers, and an output layer. In this configuration, *x*(*t*) represents the input signal of the NARNN, and 1:10 denotes the selected delay range. The value 10 below the hidden layer indicates that ten hidden neurons are used. *W* and *b* represent the connection weights and biases, respectively.

**Figure 2:**
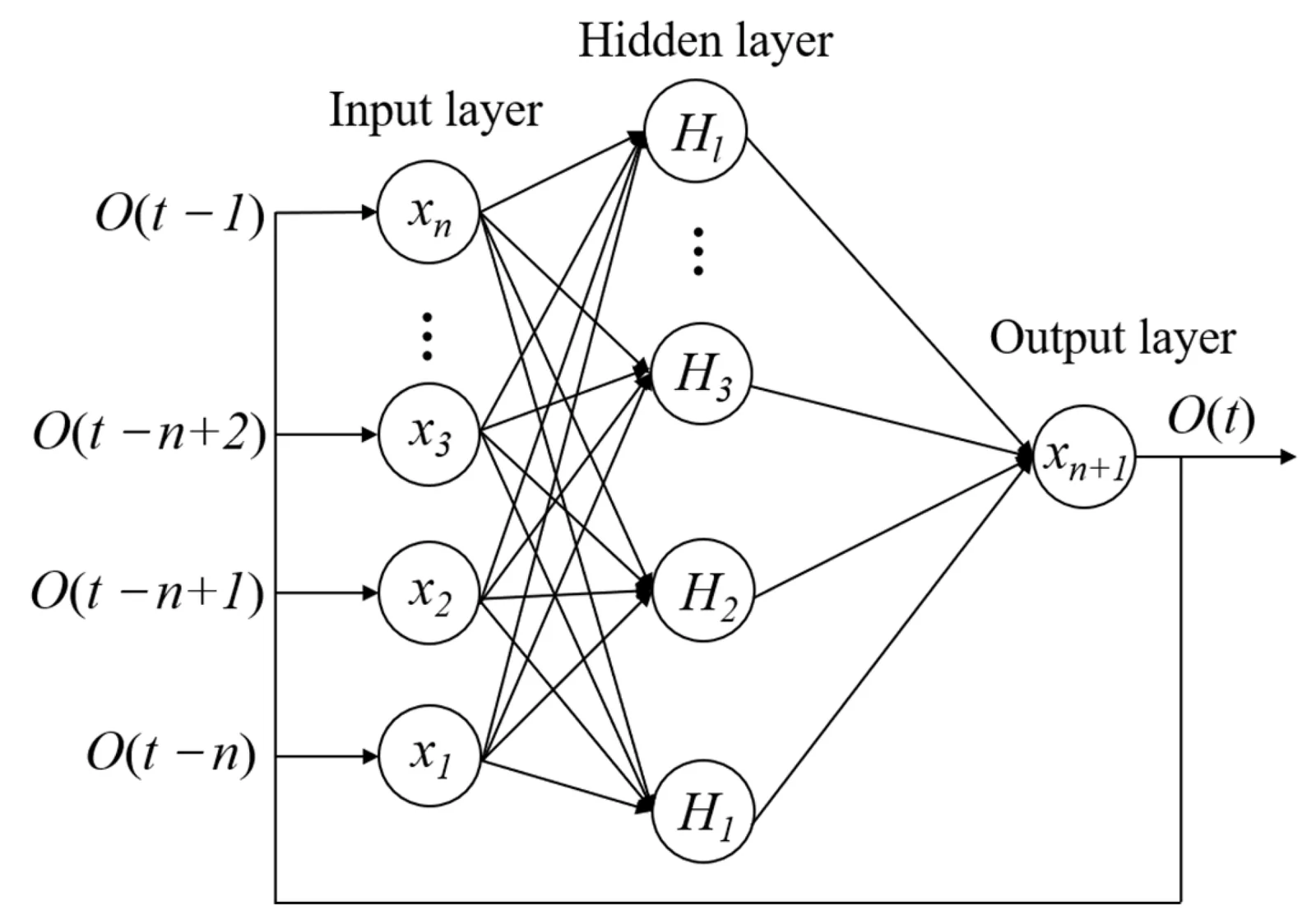
The detailed structure of the NARNN.

The detailed structure of the NARNN is presented in Figure 2. Let *x_i_* denote the sampled data point of the original signal. Given the connection weight *w_ij_* between the input node and the hidden neuron, together with the threshold *a_j_*, the output of the *j*-th hidden neuron *H_j_* can be calculated as:

(2) $$H_{j}=f(\sum_{i=1}^{n}w_{ij}x_{i}+a_{i}),j=1,2,\ldots .,l$$

where *i* is the index of the input data, *l* denotes the number of hidden neurons, and *f*(·) is the activation function of the hidden layer. Specifically, *x_i_* represents the *i*-th input datum, *w_ij_* denotes the connection weight from the *i*-th delayed signal to the *j*-th hidden neuron, and *a_j_* is the threshold of the *j*-th hidden neuron.

According to the hidden-layer output *H_j_*, the overall network output *O* can be obtained through a linear operation, which is expressed as

(3) $$O=f(\sum_{j=1}^{l}H_{j}w_{j}+b)$$

where *w_j_* represents the weight between the *j*-th hidden neuron and the output neuron, and *b* denotes the threshold of the output neuron.

### 2.2. Stacked Autoencoder

A stacked autoencoder (SAE) is a deep feature learning model constructed by stack-ing multiple autoencoders layer by layer. Its fundamental principle is to learn representative latent features from raw data through unsupervised reconstruction. A single autoencoder is mainly composed of an encoding module and a decoding module. For an input sample *x*, the encoding module transforms it into a latent representation h through a nonlinear mapping:

(4) h=f(Wx+b)

where *W* denotes the weight matrix and *b* denotes the bias vector; *f*(·) is the nonlinear activation function.

The decoding module then reconstructs the input from the latent representation:

(5) $$\hat{x}=g(W^{\prime }h+b^{\prime })$$

where *g*(·) is the decoder activation function and $\hat{x}$ denotes the reconstructed output.

The training objective is to minimize the difference between the reconstructed output and the original input. Here, the mean squared error (MSE) is used to measure the reconstruction loss:

(6) $$L=\frac{1}{N}\sum_{i=1}^{N}{\left\|x_{i}-{\hat{x}}_{i}\right\|}_{2}^{2}$$

**Figure 3:**
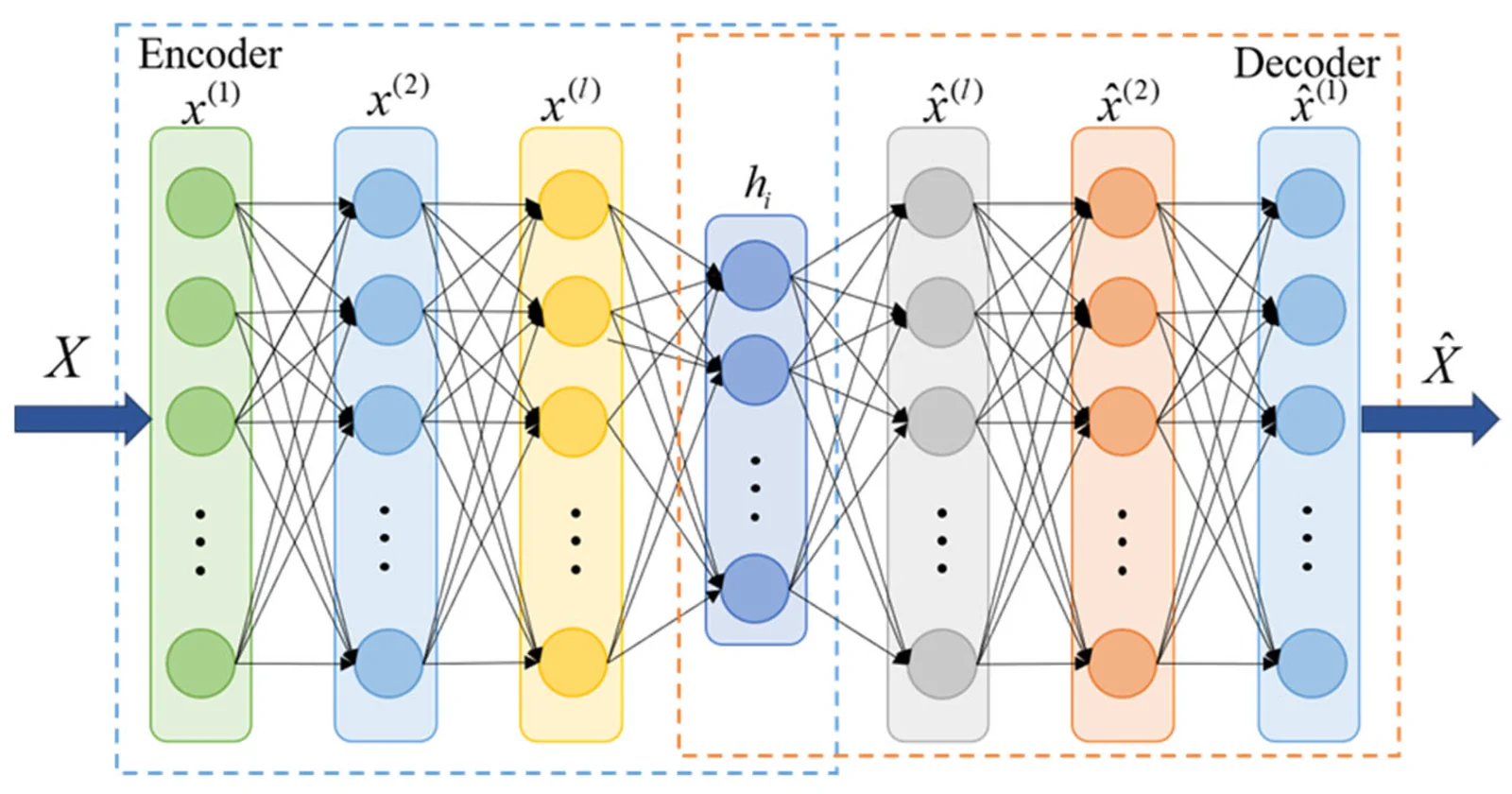
The architecture of the SAE.

As illustrated in Figure 3, the latent representation learned by each autoencoder is used as the input to the subsequent layer, forming a hierarchical feature extraction framework. Through successive encoding operations, the original high-dimensional data are gradually compressed into a low-dimensional latent space, while the decoder imposes reconstruction constraints to preserve essential information. Owing to its nonlinear representation capability, the SAE can effectively capture the complex structural characteristics embedded in vibration signals and generate compact yet informative deep feature representations for subsequent fault diagnosis.

### 2.3. Uniform Manifold Approximation and Projection

Uniform manifold approximation and projection (UMAP) is a manifold learning algorithm introduced by McInnes et al. in 2018 [25]. The method was developed for scalable dimensionality reduction and is built on concepts from Riemannian geometry and algebraic topology. Unlike t-SNE, which mainly emphasizes local structure preservation, UMAP is able to maintain neighborhood relationships while retaining certain global structural information. Therefore, it has been widely used for feature visualization and low-dimensional representation learning. Its relatively high computational efficiency also makes it suitable for processing large-scale data.

As a dimensionality reduction algorithm based on a *k*-nearest neighbor graph, UMAP mainly consists of two steps: graph construction and graph layout. In graph construction, a *k*-nearest neighbor graph is first generated using a selected distance metric to describe the local relationships between samples. The neighborhood graph is then transformed into a weighted fuzzy simplicial set, which is used to describe the manifold information contained in the high-dimensional data. In the graph layout stage, UMAP constructs a corresponding fuzzy simplicial set in the low-dimensional space and optimizes the embedding by reducing the discrepancy between the high-dimensional and low-dimensional graphs. This discrepancy is usually measured by a cross-entropy objective function. During the optimization process, samples with high similarity are encouraged to remain close to each other, whereas samples with low similarity are separated in the embedding space. The objective function is commonly optimized using stochastic gradient descent (SGD).

## 3. The Proposed Method

**Figure 4:**
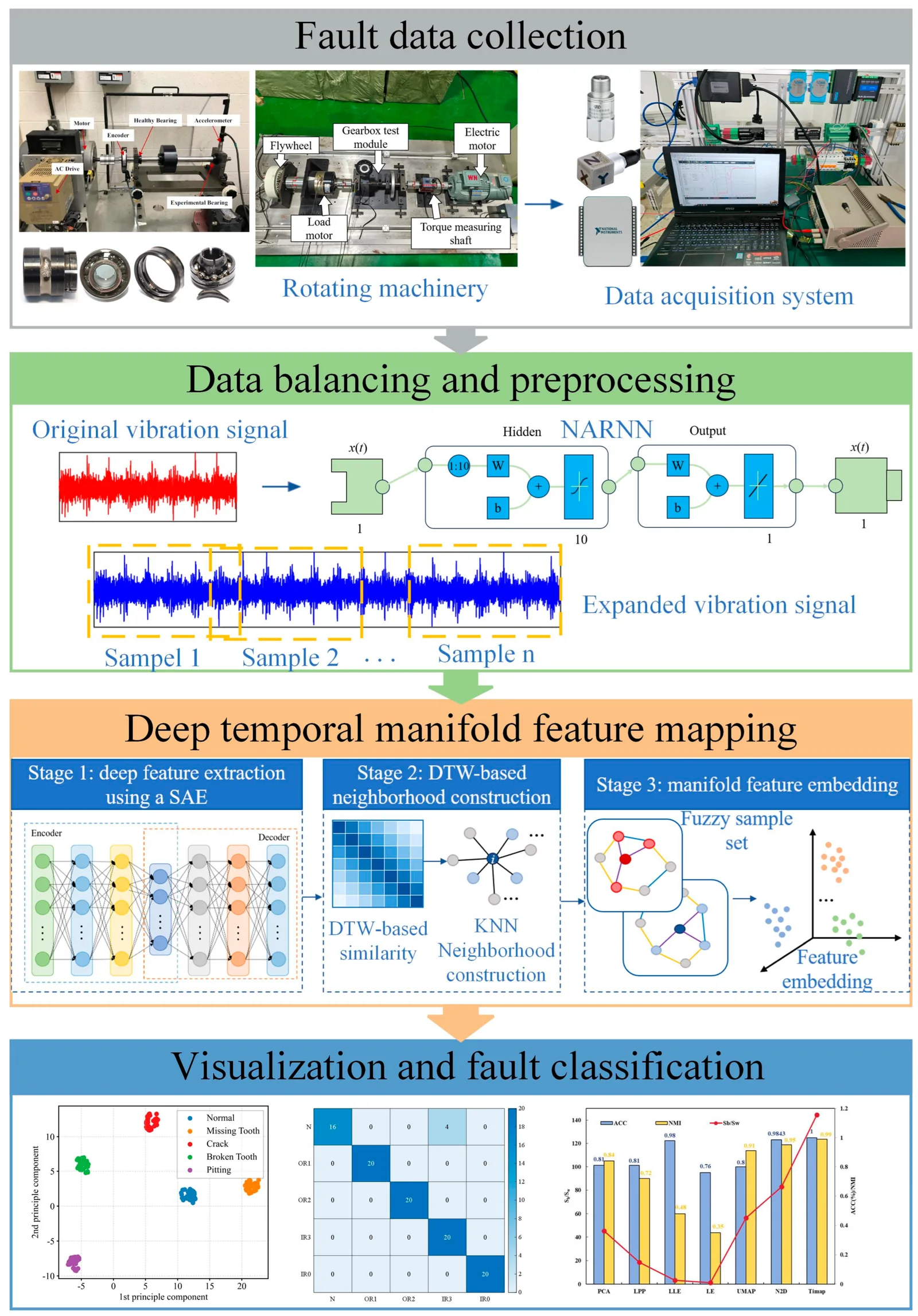
The flowchart of the proposed method.

This section presents the proposed imbalanced fault diagnosis model based on waveform-prediction augmentation and deep manifold learning. As shown in Figure 4, vibration signals from different types of rotating machinery are first collected using traditional sensors such as accelerometers. Then, an NARNN is employed to expand the minority fault signals, and the balanced vibration signals are further normalized and segmented. Meanwhile, a stacked autoencoder is employed to extract deep fault features from the preprocessed samples. Subsequently, DTW is introduced to calculate pairwise distances between the extracted features and construct a neighborhood graph for manifold approximation and projection, yielding low-dimensional representations for fault classification. Finally, the obtained features are visualized and fed into a classifier to complete fault state identification. A detailed description of the main stages is illustrated below.

### 3.1. Data Balancing and Preprocessing

Assume that the original vibration signal dataset contains *C* classes, where the length of the normal-state signal is *M_0_* and the length of the *c*-th fault signal is *M_c_*. An NARNN is employed to augment the imbalanced fault data and the length of the normal state signal is taken as the balancing reference. The *c*-th fault signal can be expressed as

(7) $$S_{c}=\{s_{1},s_{2},\ldots ,s_{M_{c}}\}$$

According to Equation (1), the NARNN establishes a nonlinear autoregressive mapping relationship based on historical time-series signals, and the prediction process can be expressed as

(8) $${\hat{s}}_{t}=f(s_{t-1},s_{t-2},\ldots ,s_{t-p})$$

where *p* denotes the time-delay order, *f*(·) is the nonlinear mapping function learned by the NARNN, and ${\hat{s}}_{t}$ represents the predicted signal. The balanced fault signal ${\tilde{S}}_{c}$ is obtained by concatenating the original fault signal with an extended sequence of length *M_0_ − M_c_*:

(9) $${\tilde{S}}_{c}=\{s_{1},s_{2},\ldots ,s_{M_{c}},{\hat{s}}_{M_{c}+1},\ldots ,{\hat{s}}_{M_{0}}\}$$

Then, Z-score normalization is applied to the balanced data of each class to minimize the effect of amplitude variations on model training and distance measurement. The normalized signal ${\bar{s}}_{i}$ is given by

(10) $${\bar{s}}_{i}=\frac{s_{i}-\mu_{i}}{\sigma_{i}}$$

where $\mu_{i}$ and $\sigma_{i}$ denote the mean and standard deviation of the corresponding sample, respectively.

Subsequently, a sliding window with length *L* and step size *r* is used to segment the normalized signals. The sample set $X=\{x_{1},x_{2},\ldots ,x_{N}\}$ ($X\in R^{N\times L}$) and the corresponding label set $Y=\{y_{1},y_{2},\ldots ,y_{N}\}$ ($Y\in R^{N\times 1}$) are obtained. Finally, the dataset is partitioned into the training set $X_{tr}=\{x_{1},x_{2},\ldots ,x_{N_{tr}}\}\in R^{N_{tr}\times L}$, the test set $X_{te}=\{x_{1},x_{2},\ldots ,x_{N_{te}}\}\in R^{N_{te}\times L}$ according to a predefined ratio, *N_tr_* and *N_te_* are the number of training and testing samples, respectively.

### 3.2. Deep Manifold Feature Mapping

After augmentation, the vibration signals of all classes maintain a consistent signal length. However, the normalized and segmented samples still contain noise and redundant information, which may degrade the stability and reliability of fault diagnosis, when they are directly fed into the subsequent classifier. To address this issue, a deep manifold feature mapping module is proposed to extract discriminative low-dimensional fault features from the balanced vibration samples. It can be seen in Figure 4 that the proposed module is implemented through three stages: deep feature extraction using an SAE, DTW-based neighborhood construction, and feature mapping and dimensionality reduction with manifold structure preservation.

#### 3.2.1. Stage 1: Deep Feature Extraction Using an SAE

First, the balanced training samples are processed by the SAE encoder for deep feature extraction. The SAE consists of an encoder and a decoder. The encoder contains three fully connected layers and maps each vibration sample to a 100-dimensional latent feature vector. ReLU activation functions are used in the hidden layers, while a sigmoid activation function is employed in the output layer of the decoder to reconstruct the input signal.

The SAE is trained in an unsupervised manner by minimizing the discrepancy between the input samples and their reconstructed outputs. In the training process, Adam is selected as the optimizer, with the learning rate, batch size, and maximum number of epochs set to 0.001, 32, and 100, respectively. To avoid overfitting, an early-stopping strategy is introduced. Specifically, if the validation loss fails to decrease for five consecutive epochs, the training procedure is terminated, and the model parameters with the minimum validation loss are retained for subsequent feature extraction.

After training, the decoder is removed, and only the encoder is retained to map the balanced training samples into a low-dimensional latent feature space. The obtained deep latent features are denoted as

(11) $$H=\{h_{1},h_{2},\ldots ,h_{N_{tr}}\}\in R^{N_{tr}\times L_{h}}$$

where *L_h_* denotes the dimensionality of the SAE-extracted latent features. In this study, *L_h_* is set to be 100. These compact representations reduce redundancy information and provide the input for the subsequent DTW-based neighborhood construction.

#### 3.2.2. Stage 2: DTW-Based Neighborhood Construction

Dynamic time warping (DTW) is a path-based distance measure that quantifies sample dissimilarity by identifying the minimum-cost alignment path through a local cost matrix. It first constructs a Euclidean distance matrix and then applies dynamic programming to identify the optimal warping path by backtracking through this matrix. In the proposed framework, DTW is used to calculate pairwise distances between the SAE-derived features. The resulting distances are then used to identify neighboring samples and construct the neighborhood graph for subsequent manifold embedding.

Let $X=\{x_{1},x_{2},\ldots ,x_{n}\}$ and $Y=\{y_{1},y_{2},\ldots ,y_{m}\}$ be two time-series signals with lengths *n* and *m*, respectively, where *i* and *j* index the elements of *X* and *Y*, respectively.

First, an *n* × *m* local cost matrix ***d*** is constructed. Each element *d*(*i*,*j*) denotes the local matching cost between *x_i_* and *y_j_*, which is calculated using the Euclidean distance as follows:

(12) $$d(i,j)=\sqrt{{\left(x_{i}-y_{j}\right)}^{2}}$$

Then, the cumulative cost matrix is computed recursively using Bellman’s recursion. Therefore, the DTW distance between *X* and *Y* can be obtained from the minimum cumulative matching cost as follows:

(13) $$D(i.j)=d(i,j)+\min\left\{\begin{array}{c} D(i-1,j)\\ D(i,j-1)\\ D(i-1,j-1) \end{array}\right.$$

(14) DTW(X,Y)=D(n,m)

where *D*(*i*,*j*) denotes the minimum cumulative matching cost from the starting point (1,1) to the current position (*i*,*j*), and *D*(*n*,*m*) represents the final DTW distance between the two sequences.

**Figure 5:**
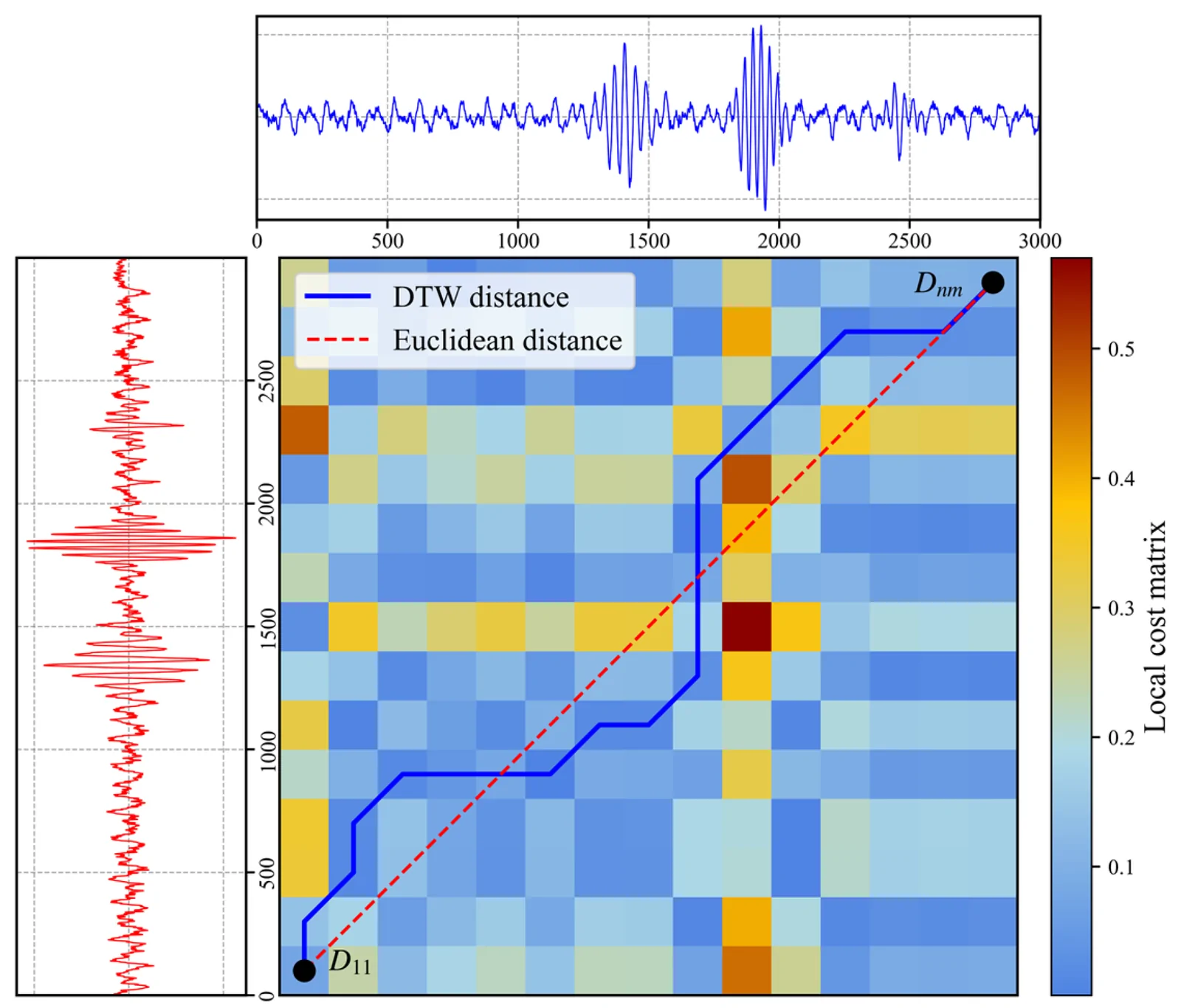
Comparison between DTW distance and Euclidean distance.

Figure 5 illustrates the computational differences between DTW and Euclidean distance. The central grid represents the local cost matrix, where dark red and light blue regions indicate high and low local costs, respectively. The red dashed diagonal denotes the fixed one-to-one correspondence used by Euclidean distance, whereas the blue curve shows the minimum cumulative-cost path obtained by dynamic programming. Unlike the fixed diagonal correspondence, DTW provides a more flexible matching scheme by allowing non-diagonal transitions, causing the two distance measures to produce different pairwise distances and neighborhood relationships for the same feature representations.

Direct pairwise DTW calculations can become computationally expensive for large-scale vibration datasets. Therefore, FastDTW is considered an optional approximation for efficiently computing pairwise dissimilarities between SAE-derived features, particularly when extending DMAP to large-scale datasets or online monitoring applications [32].

Given two latent feature vectors $h_{i}=\{h_{i1},h_{i2},\ldots ,h_{iL_{h}}\}$ and $h_{j}=\{h_{j1},h_{j2},\ldots ,h_{jL_{h}}\}$, their FastDTW distance is defined as

(15) $$d_{ij}=FastDTW(h_{i},h_{j})=D_{ij}(L_{h},L_{h})$$

where *D_ij_*(*L_h_*, *L_h_*) is the terminal element of the cumulative cost matrix, representing the minimum accumulated matching cost corresponding to the optimal warping path from the starting point (1,1) to (*L_h_*, *L_h_*). The recursive calculation of the cumulative cost matrix is given in Equations (5) and (6). The similarity matrix is obtained as

(16) $$D_{\mathrm{FastDTW}}=\left[\begin{array}{cccc} 0 & d_{12} & \ldots & d_{1N_{tr}}\\ d_{21} & 0 & \ldots & d_{2N_{tr}}\\ \vdots & \vdots & \ddots & \vdots \\ d_{N_{tr}1} & d_{N_{tr}2} & \ldots & 0 \end{array}\right]$$

where the element *d_ij_* denotes the DTW distance between sample *h_i_* and sample *h_j_*. Based on this distance matrix, the *k*-nearest neighborhood $N_{k}({\hat{x}}_{i})$ of each sample is constructed.

#### 3.2.3. Stage 3: Manifold Feature Embedding

For any sample *h_i_* and its neighboring sample, the directed fuzzy membership degree is defined as

(17) $$\mu ({\hat{x}}_{i},{\hat{x}}_{j})=\exp\left(-\frac{\max(0,d({\hat{x}}_{i},{\hat{x}}_{j})-\rho_{i})}{\sigma_{i}}\right)$$

where $d({\hat{x}}_{i},{\hat{x}}_{j})$ is the DTW distance between point *x_i_* and *x_j_*; *ρ_i_* is the local connectivity constraint used to ensure that points connect to at least one data point with an edge of weight 1; and *σ_i_* is the normalization factor that controls the smoothness of the neighbors. The calculations of *ρ_i_* and *σ_i_* are derived from the following:

(18) $$\rho_{i}=\min\{d(x_{i},x_{ij})|1\leq j\leq k,d(x_{i},x_{ij})>0\}$$

(19) $$\sum_{j=1}^{k}\exp(\frac{-\max(0,d(x_{i},x_{ij})-\rho_{i})}{\sigma_{i}})=\log_{2}(k)$$

Since the neighborhood relationship between samples is directional and usually asymmetric, a fuzzy set operation is adopted to symmetrize the directed fuzzy membership degrees, thereby obtaining an undirected weighted neighborhood graph:

(20) $$B_{ij}=\mu ({\hat{x}}_{i},{\hat{x}}_{j})+\mu ({\hat{x}}_{j},{\hat{x}}_{i})-\mu ({\hat{x}}_{i},{\hat{x}}_{j})\mu ({\hat{x}}_{j},{\hat{x}}_{i})$$

where *B_ij_* denotes the fuzzy adjacency weight between samples in the high-dimensional space. The fuzzy adjacency weights of all samples constitute a high-dimensional weighted graph *G* = (*V*,*E*,*B*), where *V* is the sample set, *E* is the edge set, and *B* is the edge weight matrix.

In the graph layout stage, assume that the representation of samples in the low-dimensional space is $Z=\{z_{1},z_{2},\ldots ,z_{N_{tr}}\}\in R^{N_{tr}\times d}$, where *d* is the embedding dimensionality. The fuzzy membership degree between samples in the low-dimensional space is defined as

(21) $$\nu_{ij}={\left(1+a\|z_{i}-z_{j}{\|}^{2b}\right)}^{-1}$$

where $\left\|z_{i}-z_{j}\right\|$ denotes the distance between two points in the low-dimensional space, and *a* and *b* are hyperparameters used to control the scale and density distribution of the low-dimensional embedding. These two parameters are determined by curve fitting according to the preset parameters *d_min_* and *s*, *d_min_* denotes the minimum distance between sample points in the low-dimensional space, and *s* represents the distribution scale of the low-dimensional embedding.

During the calculation, the target distance decay function ϕ(r) is first constructed. When the distance between two low-dimensional points is smaller than *d_min_*, a high similarity is maintained; when the distance is larger than *d_min_*, the similarity gradually decays as the distance increases:

(22) $$\phi (r)=\left\{\begin{array}{ll} 1, & 0\leq r<d_{\min}\\ \exp\left(-\frac{r-d_{\min}}{s}\right), & r\geq d_{\min} \end{array}\right.$$

However, the function actually used in the dimensionality reduction optimization process is

(23) $$\nu (r;a,b)=\frac{1}{1+ar^{2b}}$$

Therefore, nonlinear least-squares fitting is performed to make v(r;a,b) approximate the target function ϕ(r) as closely as possible, thereby converting the preset physical parameters *d_min_* and *s* into the hyperparameters *a* and *b* used in the optimization function:

(24) $$(a^{*},b^{*})=\arg\;\min_{a,b}\sum_{r}{\left[\phi (r)-\frac{1}{1+ar^{2b}}\right]}^{2}$$

In this study, *d_min_* = 0.1 and *s* = 1.0 are set; the fitted hyperparameters are *a* ≈ 1.6 and *b* ≈ 0.9.

The neighborhood structure measured by DTW in the original feature space is preserved by optimizing the cross-entropy loss between the fuzzy graph in the high-dimensional space and its counterpart in the low-dimensional space. The optimization objective can be written as:

(25) $$Loss=\sum_{i,j}\left[B_{ij}\log(\nu_{ij})+(1-B_{ij})\log(1-\nu_{ij})\right]$$

The test samples are input into the trained SAE encoder to obtain their deep feature representations. The DTW-based distances between the test latent features and the training latent features are then computed. Using the obtained distance matrix, the nearest training neighbors of each test sample are searched. Subsequently, each test sample is embedded into the learned low-dimensional space according to its neighborhood relationship with the training samples. The resulting low-dimensional test features are denoted as *Z_te_*.

The pseudo-code of the proposed DMAP algorithm is shown in Algorithm 1.
**Algorithm 1:** Deep manifold feature mapping (DMAP) **Input:** the balanced training samples *X_tr_*, test samples *X_te_*, number of neighbors *k*, embedding dimension *d*, and SAE parameters Θ; **Output:** the low-dimensional features *Z_tr_* and *Z_te_*;
1. Initialize the SAE parameters Θ; 2. Train the SAE by minimizing the reconstruction loss; 3. Obtain the deep hidden features of training samples: *H_tr_* = Encoder(*X_tr_*); 4. Obtain the deep hidden features of test samples: *H_te_* = Encoder(*X_te_*); 5. Calculate the DTW-based distance between latent features: *d_ij_* = DTW(*h_i_*, *h_j_*); 6. Construct the *k*-nearest neighbor graph based on the FastDTW distance; 7. Compute the fuzzy membership strength of each edge; 8. Construct the high-dimensional fuzzy graph; 9. Initialize the low-dimensional embedding *Z_tr_*; 10. **for** *i* in epochs do:
11.    Optimize the cross-entropy loss between the high-dimensional and low-dimensional fuzzy graphs;
12.    Update the low-dimensional embedding *Z_tr_*; 13. **end for** 14. Project the test features *H_te_* into the learned low-dimensional space; 15. **return** *Z_tr_* and *Z_te_*.

## 4. Case Study

### 4.1. Case Study 1: Machinery Failure Prevention Technology Society (MFPT) Bearing Dataset

#### 4.1.1. Dataset Description

The public bearing dataset collected from the Machinery Failure Prevention Technology Society [33] was used in this experiments to evaluate the effectiveness of the proposed method. The detailed description of the considered rolling bearing vibration signals conditions in the section is provided in Table 1. The input shaft rate was 25 Hz.

The raw one-dimensional vibration signal was first divided into separate training and testing segments, and then partitioned into samples of 4000 points each. To increase the number of samples, overlapping sampling was employed, with each sample overlapping by 3000 points.

Four datasets were constructed with the processed samples: a balanced dataset (Dataset A), a moderately imbalanced dataset (Dataset B), a severely imbalanced dataset (Dataset C), and an extremely imbalanced dataset (Dataset D). The training samples comprised both original measured samples and NARNN-generated samples. Dataset A contained only original samples, whereas the minority classes in Datasets B, C, and D were supplemented with generated samples to alleviate class imbalance. The normal-class samples remained entirely original, and all testing samples were obtained exclusively from the original measured vibration signals. The detailed numbers of original and generated samples are presented in Table 2.

#### 4.1.2. The Parameter Setting and Performance of the NARNN

The NARNN has two key parameters to be determined: the delay order and the number of hidden layer nodes (NHLN). The former specifies how many time steps of past data are used for prediction, while the latter determines the scale of the hidden layer, which in turn governs the nonlinear fitting capacity and complexity of the model.

An excessively large delay order can result in a high input dimensionality, thereby increasing training difficulty and the risk of overfitting, whereas an overly small delay order may fail to capture temporal correlations of time sequences. For the NHLN, an excessive number of nodes increases the training cost, while an insufficient number of nodes may weaken the fitting ability of the model. Therefore, selecting suitable values for these parameters is necessary to the performance of the NARNN.

In this study, MSE and training time were adopted as evaluation criteria. The MSE was expressed as:

(26) $$MSE=\frac{1}{n}{\sum_{t=1}^{n}\left(y_{t}-{\overset{\wedge }{y}}_{t}\right)}^{2}$$

**Figure 6:**
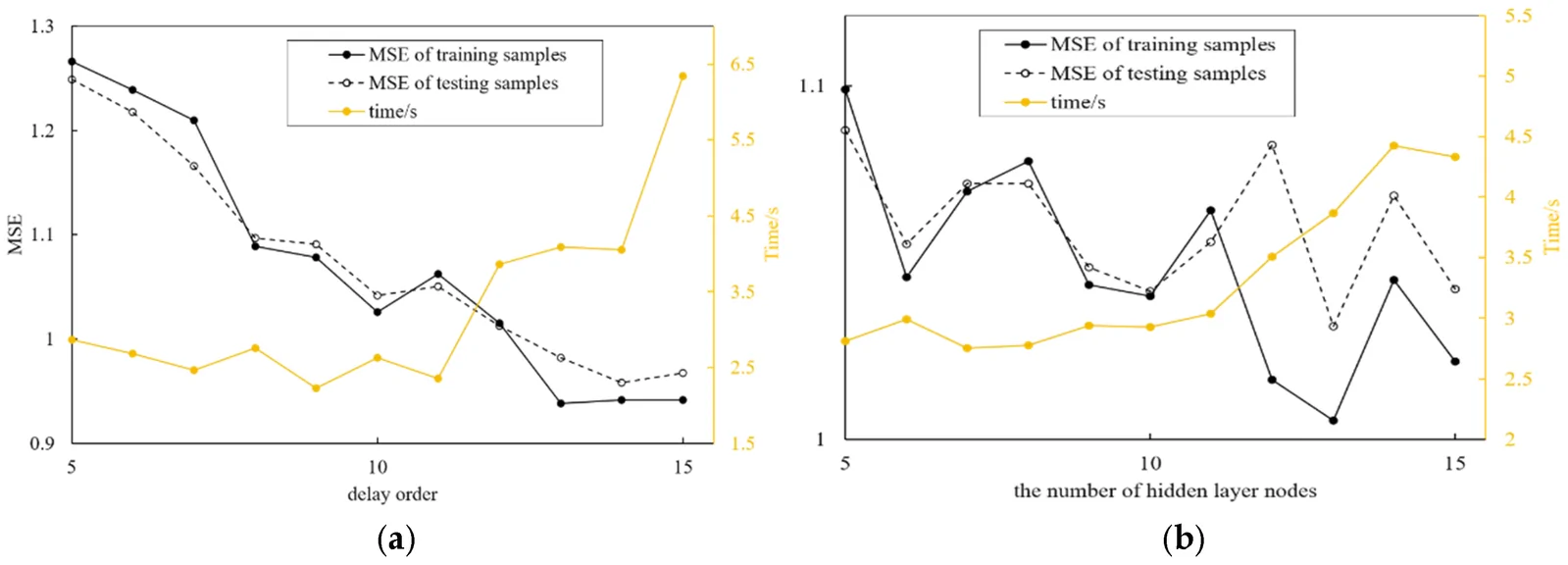
MSE under different parameter settings: (**a**) the selection of optimal delay order; (**b**) the selection of the optimal NHLN.

The vibration signal from IR3 was used as a representative example to illustrate the parameter-selection process. The NHLN was first fixed at 10, and the delay order was varied from 5 to 15. Then, with the delay order determined as 10, the NHLN was optimized within the range of 5 to 15. From Figure 6a, it can be seen that the MSE of both training and testing samples decreases with an increase in the delay order, while the computational time correspondingly increases. Similarly, the curve in Figure 6b exhibits a comparable variation trend. Therefore, as a trade-off, both the delay order and the NHLN were set to 10 in the subsequent experiments.

#### 4.1.3. Performance of the Proposed Method

**Figure 7:**
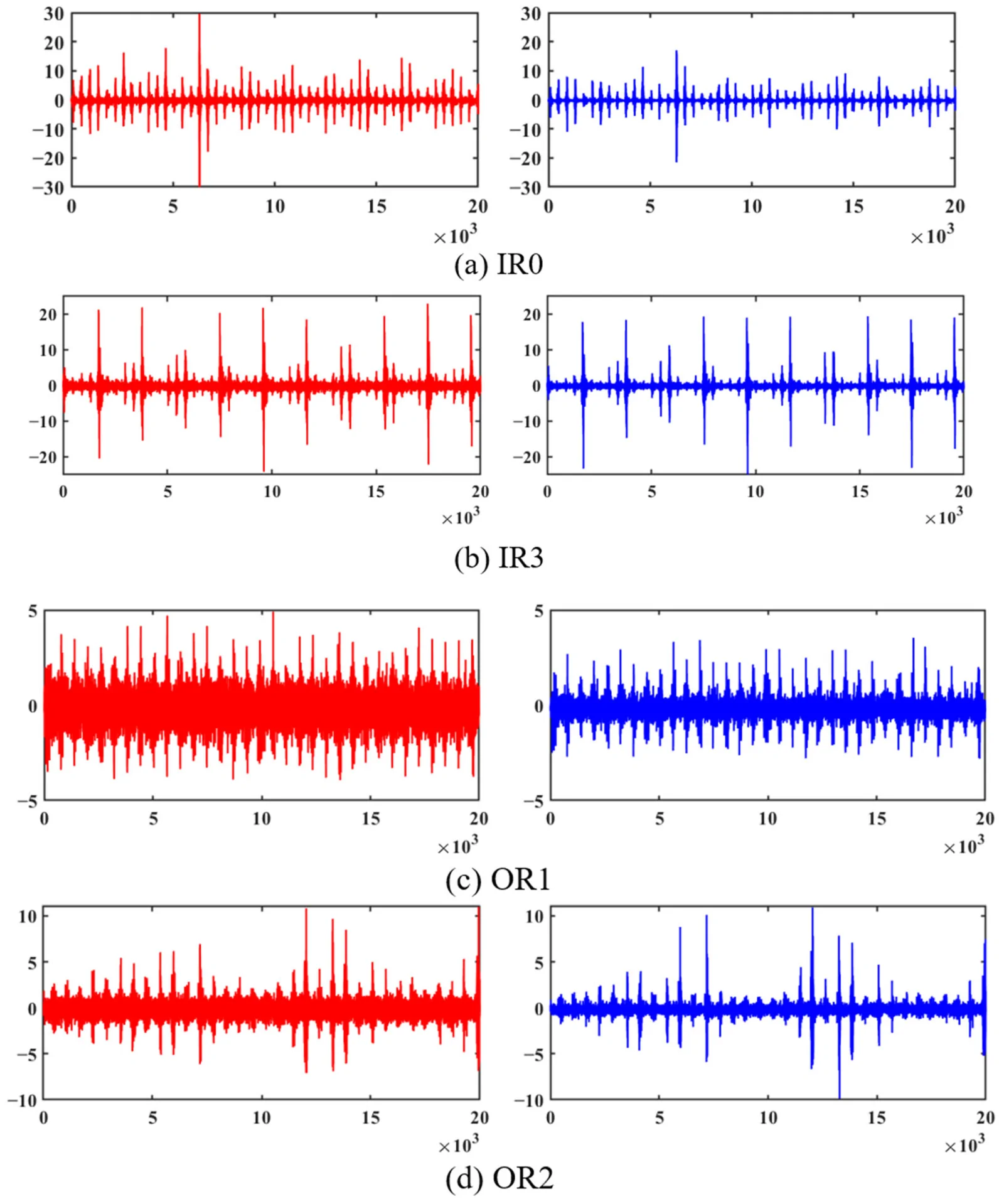
The comparison between original signals and NARNN-generated signals.

**Figure 8:**
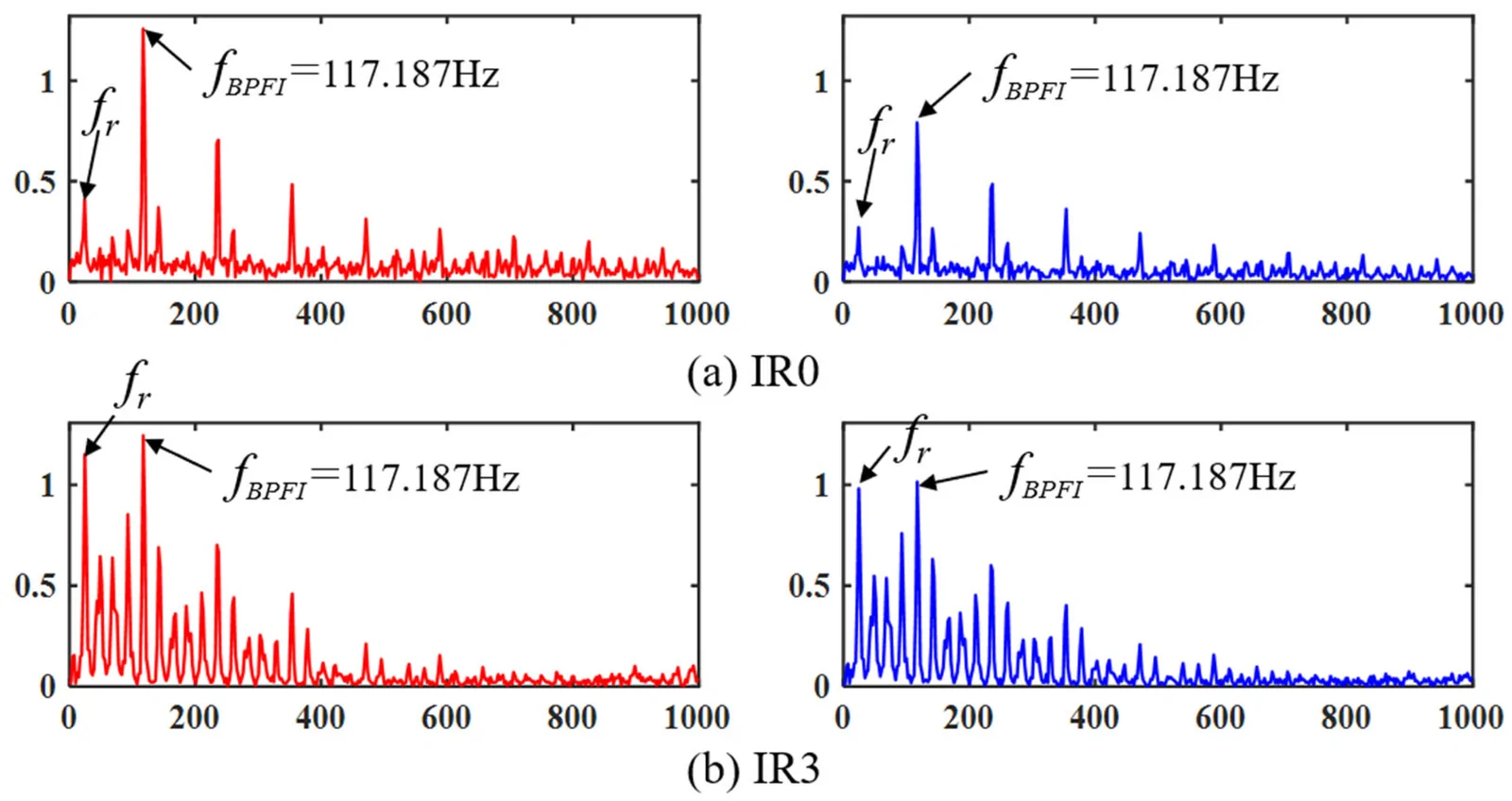

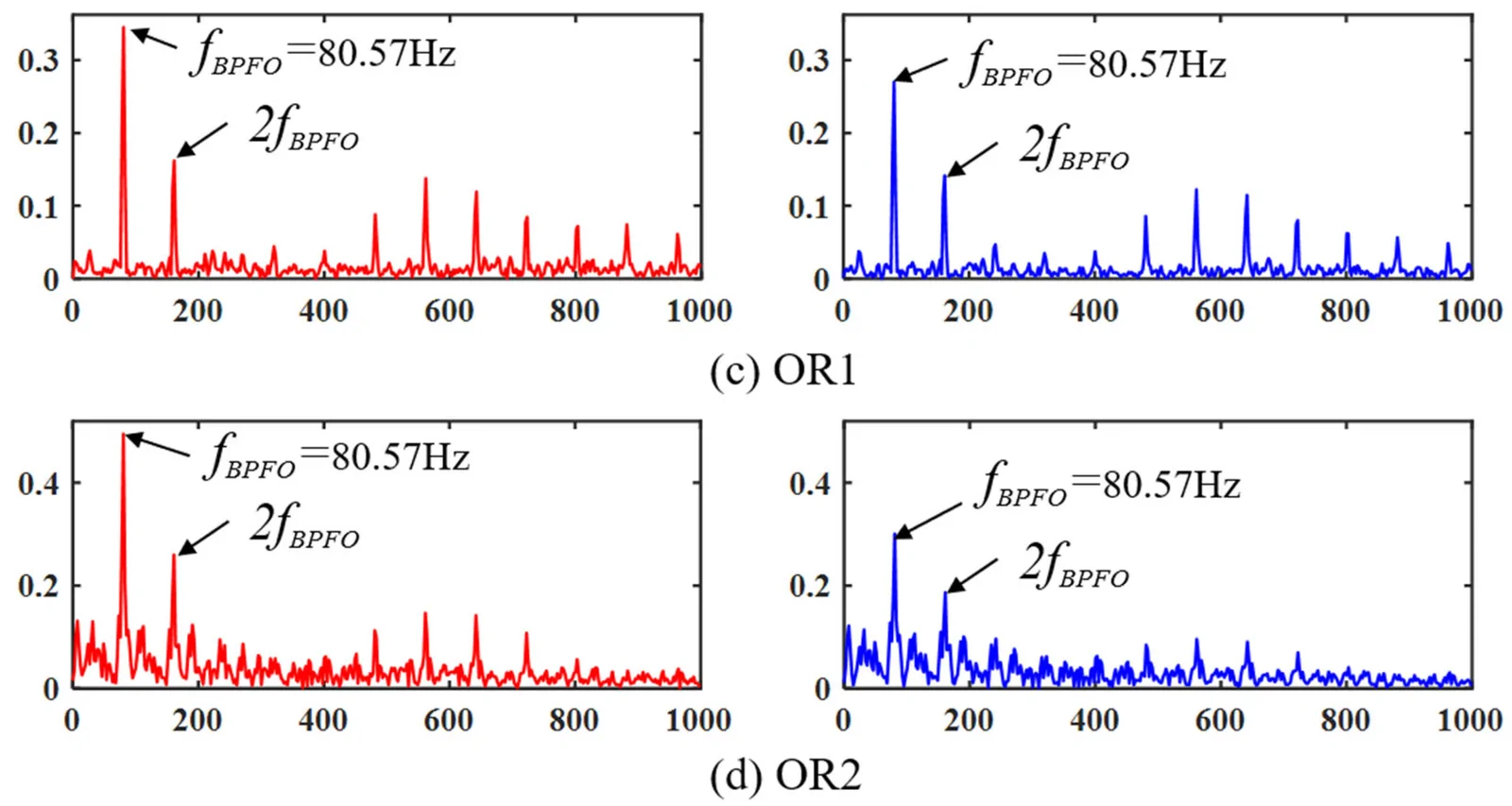
The envelope spectrum comparison between original signals and NARNN-generated signals.

The quality of the NARNN-generated samples was first examined by comparing the generated part with the corresponding portion of the original measured signal at the same temporal positions. The comparison results are presented in Figure 7, where the red curves indicate the original signals and the blue curves indicate the generated signals. The generated signals exhibit trends and impact characteristics similar to those of the original signals. Moreover, their envelope spectra retain the main fault characteristic frequencies as shown in Figure 8. Specifically, clear peaks appear at *f_BPFI_* = 117.187 Hz for the inner-race faults and at *f_BPFO_* = 80.57 Hz and its second harmonic for the outer-race faults. Although slight differences in spectral amplitudes are observed, the locations and overall distributions of the principal peaks remain consistent, indicating that the generated samples preserve the essential fault-related features of the original signals.

To verify the effectiveness of the proposed method for fault diagnosis under imbalanced data scenarios, the balanced data generated by the NARNN were input into DMAP for feature extraction and dimensionality reduction, and the extracted features were subsequently fed into the KNN classifier to obtain the diagnostic results. For comparison, the imbalanced data were directly subjected to the same feature extraction and classification. The results are shown in Table 3. The mean accuracy, Macro-F1, and G-mean obtained from five independent experiments were adopted to comprehensively evaluate the diagnostic performance. Accuracy measures the overall proportion of correctly classified samples, whereas Macro-F1 and G-mean give equal consideration to each class and are therefore more suitable for evaluating classification performance under class imbalance.

(27) $$Accuracy=\frac{\sum_{i=1}^{C}TP_{i}}{N}$$

(28) $$Macro-F1=\frac{1}{C}\sum_{i=1}^{C}\frac{2P_{i}R_{i}}{P_{i}+R_{i}}$$

(29) $$G-mean={\left(\prod_{i=1}^{C}R_{i}\right)}^{1/C}$$

where *C* denotes the number of classes and *N* is the total number of testing samples. *P_i_* and *R_i_* denote the precision and recall of the *i*-th class, respectively, which were calculated as

(30) $$P_{i}=\frac{TP_{i}}{TP_{i}+FP_{i}}$$

(31) $$R_{i}=\frac{TP_{i}}{TP_{i}+FN_{i}}$$

where *TP_i_*, *FP_i_*, and *FN_i_* represent the true positives, false positives, and false negatives for the *i*-th class, respectively. G-mean is sensitive to poor recognition of minority classes; a low recall for any class will considerably reduce the overall G-mean.

It can be seen from Table 3 that the proposed feature extraction method effectively captures discriminative fault features from the original balanced Dataset A, achieving an average accuracy of 98.20% ± 2.49%. However, as the degree of class imbalance increases, the accuracy decreases to 95.60%, 90.60%, and 67.00% for Datasets B, C, and D, respectively. This degradation occurs because the neighborhood structure constructed from imbalanced data is dominated by majority-class samples. Consequently, the intrinsic structures of minority classes may be insufficiently represented, reducing interclass separability and subsequent classification performance. After NARNN-based data augmentation, the accuracies of Datasets B, C, and D increased by 2.40%, 4.00%, and 10.20%, respectively. Similar improvements were observed in Macro-F1 and G-mean. In particular, for Dataset D, Macro-F1 increased from 66.10% to 76.07%, while G-mean increased from 63.14% to 74.14%, indicating improved recognition of minority classes. Although performance under extreme imbalance remained lower than that on the balanced Dataset A, the consistent improvements across the diagnostic results demonstrate that the NARNN effectively alleviates the effects of class imbalance and improves the robustness of the proposed diagnostic method.

**Figure 9:**
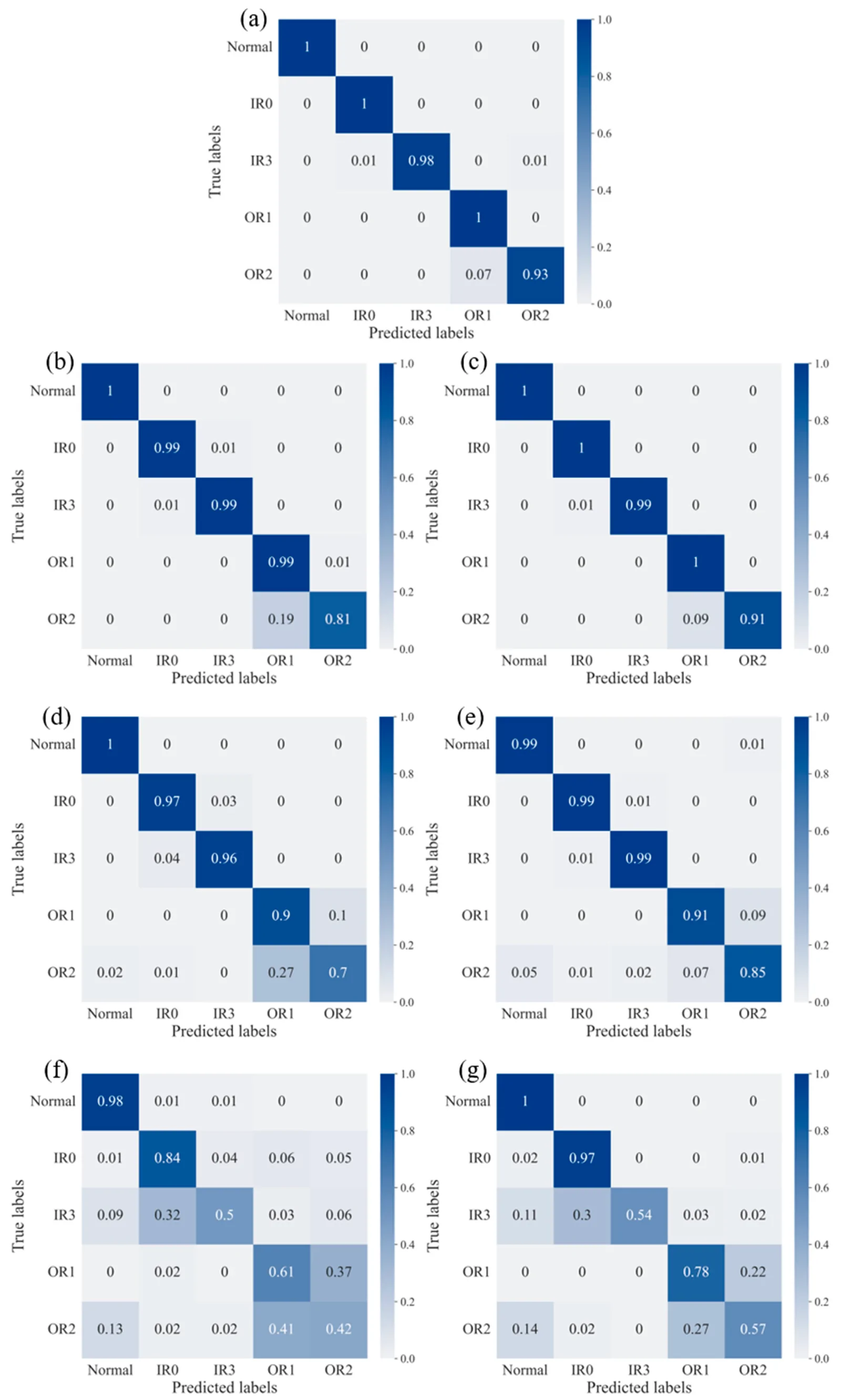
The confusion matrix of the diagnosis results under different datasets: (**a**) Dataset A; (**b**) imbalanced Dataset B; (**c**) NARNN-balanced Dataset B; (**d**) imbalanced Dataset C; (**e**) NARNN-balanced Dataset C; (**f**) imbalanced Dataset D; (**g**) NARNN-balanced Dataset D.

The corresponding confusion matrix is shown in Figure 9, where each element represents the recall for the corresponding class. By predicting and supplementing the minority-class signals using the NARNN, the manifold features of minority classes are reconstructed, thereby effectively addressing the fault diagnosis problem under imbalanced data conditions.

#### 4.1.4. Comparison with Other Diagnostic Methods

To further validate the superiority of the proposed method, comparative experiments were performed on Dataset B from two aspects: data augmentation and feature extraction. Specifically, the feature extraction module was compared with PCA [18], LLE [20], LE [21], LPP [22], UMAP [25], and N2D [26], The detailed parameter settings are shown in Table 4.

In addition to the index mentioned above, the normalized mutual information (NMI) [26] and the inter-class/intra-class distance ratio *S_b_*/*S_w_* [34] were adopted to further evaluate the classification consistency and feature separability of the proposed method.

The NMI measures the consistency between the model-predicted labels and the actual labels from the perspective of information entropy. Assume that the set of true labels in the dataset is denoted as *Y*, and the clustering result predicted by the model is denoted as *C*. The NMI is calculated as follows. The value of NMI lies between 0 and 1, where a larger value means that the predicted clustering distribution is more consistent with the real label distribution.

(32) $$NMI(Y,C)=\frac{2I(Y;C)}{H(Y)+H(C)}$$

(33) $$I(Y;C)=\sum_{y\in Y}\sum_{c\in C}P(y,c)\log(\frac{P(y,c)}{P(y)P(c)})$$

where *I(Y*;*C)* represents the mutual information; *H(Y)* and *H(C)* are the corresponding information entropies used for normalization for comparison among different models; *P(y)* and *P(c)* denote the probabilities that a sample belongs to the true class *y* and the predicted class *c*, respectively; and *P(y,c)* denotes the joint probability that a sample belongs to both classes.

The intra-class scatter *S_w_* measures the compactness of samples within the same class, while the inter-class scatter *S_b_* reflects the separability among different classes. Therefore, a larger *S_b_*/*S_w_* ratio indicates stronger discriminative ability of the extracted features. The corresponding calculations are as follows:

(34) $$S_{b}={\sum_{c=1}^{N_{c}}\left(u_{\alpha }^{c}-{\bar{u}}_{\alpha })(u_{\alpha }^{c}-{\bar{u}}_{\alpha }\right)}^{T}$$

(35) $$S_{w}={\sum_{c=1}^{N_{c}}\sum_{\alpha_{k}\in c}\left(\alpha_{k}-u_{\alpha }^{c})(\alpha_{k}-u_{\alpha }^{c}\right)}^{T}$$

where *α_k_*(*k* = 1,2,…,*d*) denotes the *k*-th samples, $u_{\alpha }^{c}$ is the mean vector of class c, and ${\bar{u}}_{\alpha }$ is the global mean vector of all samples.

**Figure 10:**
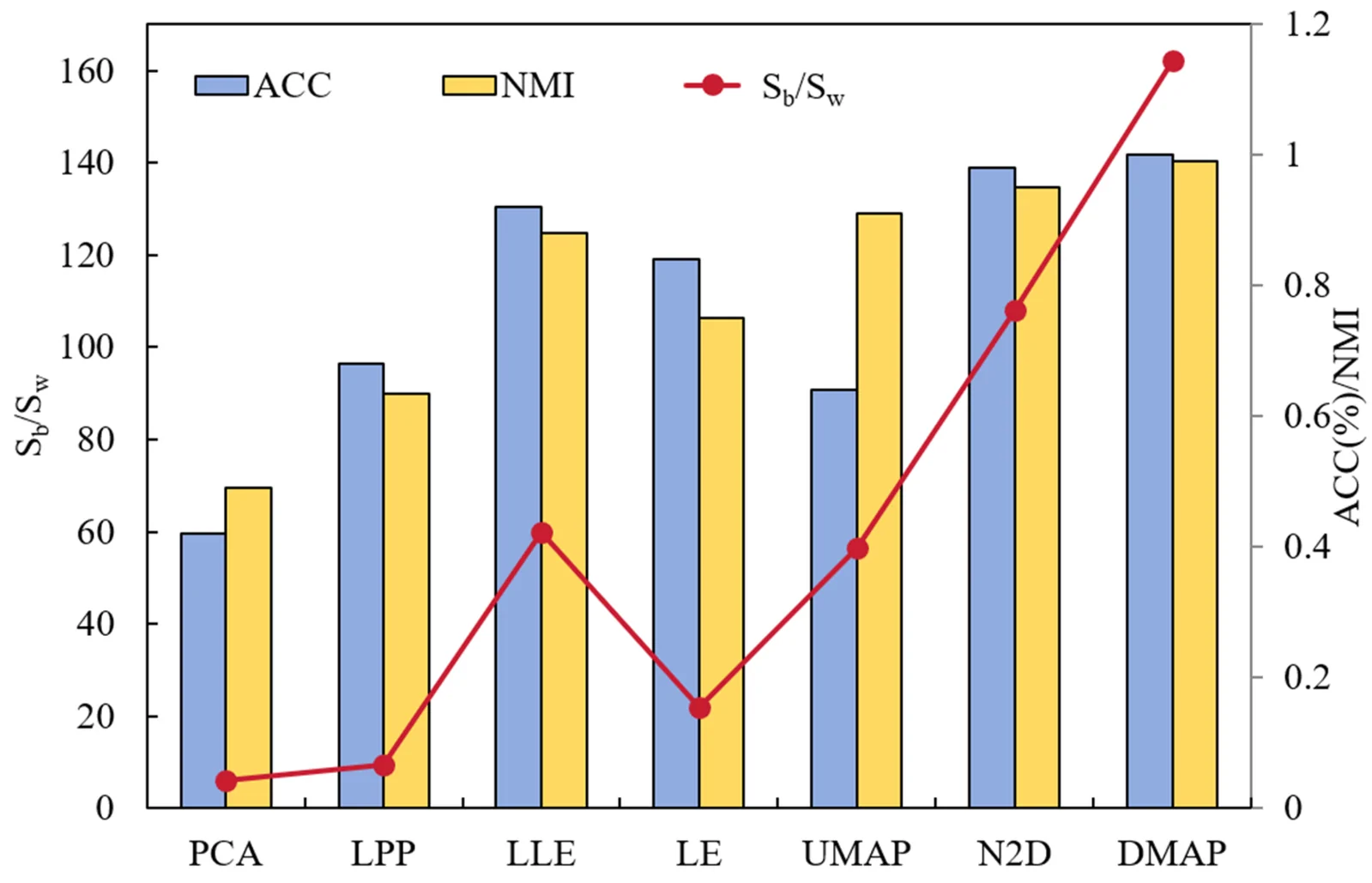
Comparison results between different feature extraction methods.

Figure 10 presents the quantitative results of different feature extraction and dimensionality reduction algorithms. DMAP achieves higher ACC and NMI values than the other methods, indicating that it can produce more reliable diagnostic results. In addition, the *S_b_*/*S_w_* ratio of DMAP is significantly higher than those of the other six feature extraction algorithms demonstrates that the features extracted by DMAP exhibit stronger intra-class compactness and inter-class separability. These results confirm that DMAP can extract more discriminative fault features.

**Figure 11:**
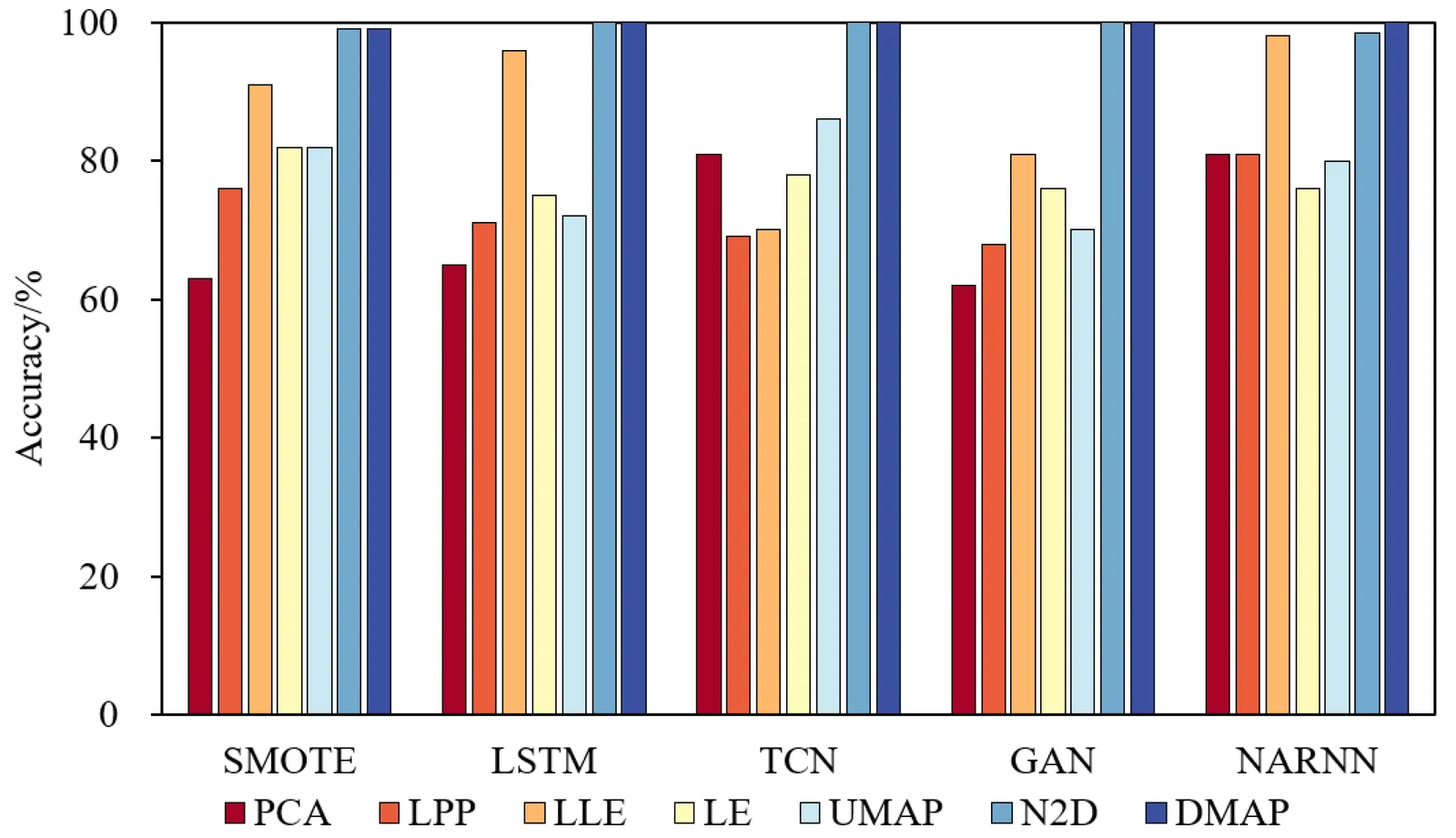
Diagnostic accuracy under different data augmentation methods.

Furthermore, the data augmentation module was compared with SMOTE [35], long short-term memory (LSTM) [36], temporal convolutional network (TCN) [37], and GAN [38], and the detailed parameter settings are shown in Table 5. The diagnostic accuracy is recorded in Figure 11. Meanwhile, the time consumption required to augment the IR0 data within Dataset B using various data augmentation algorithms is shown in Table 6.

It can be observed that DMAP consistently maintains high diagnostic accuracy across different data-generation methods, demonstrating its effectiveness in extracting discriminative fault features. Meanwhile, employing the NARNN for signal prediction and augmentation of minority classes yields superior overall performance compared to the traditional SMOTE interpolation, the recursive-structure-based methods LSTM, and other generative architectures including TCN and GAN. This superiority is particularly evident when utilizing nonlinear manifold learning algorithms (e.g., LLE and LE). LSTM tends to retain long-term memory, and when predicting signals, it more closely retains the overall trend of the samples while ignoring local details. TCN and GAN are prone to overfitting and mode collapse in small-sample scenarios, resulting in a decline in the quality of generated samples and a consequent decrease in classification accuracy. However, the signals generated by the NARNN effectively preserve temporal continuity and class-discriminative characteristics, enabling NARNN-based augmentation to achieve slightly higher accuracy than SMOTE even when combined with traditional feature extraction and dimensionality-reduction methods. SMOTE requires only 0.019 s and therefore provides the highest augmentation efficiency; however, as an interpolation-based method, it does not explicitly model the temporal dependencies of vibration signals. In comparison with the other deep-learning-based generation methods, the NARNN requires the least computation time (3.31 s), demonstrating a favorable balance between diagnostic performance and computational cost.

#### 4.1.5. Ablation Experiments

To evaluate the contribution of each component in the proposed model, ablation experiments based on Dataset B were conducted by removing key modules, including NARNN-based data balancing, SAE-based deep feature extraction, and DTW-based manifold mapping. The specific settings of the ablation models are presented in Table 7. M1 represents the complete proposed model; M2 removes the data balancing module based on M1; M3 removes the SAE module; M4 removes the DTW-UMAP module; M5 replaces DTW-UMAP in M1 with standard UMAP based on the Euclidean distance, while retaining both NARNN and SAE; in M6, the dimensionality-reduction module is removed, and the augmented balanced data are directly fed into a DTW-based KNN classifier. M7 is constructed based on M3 by further replacing DTW-UMAP with standard UMAP based on the Euclidean distance.

**Figure 12:**
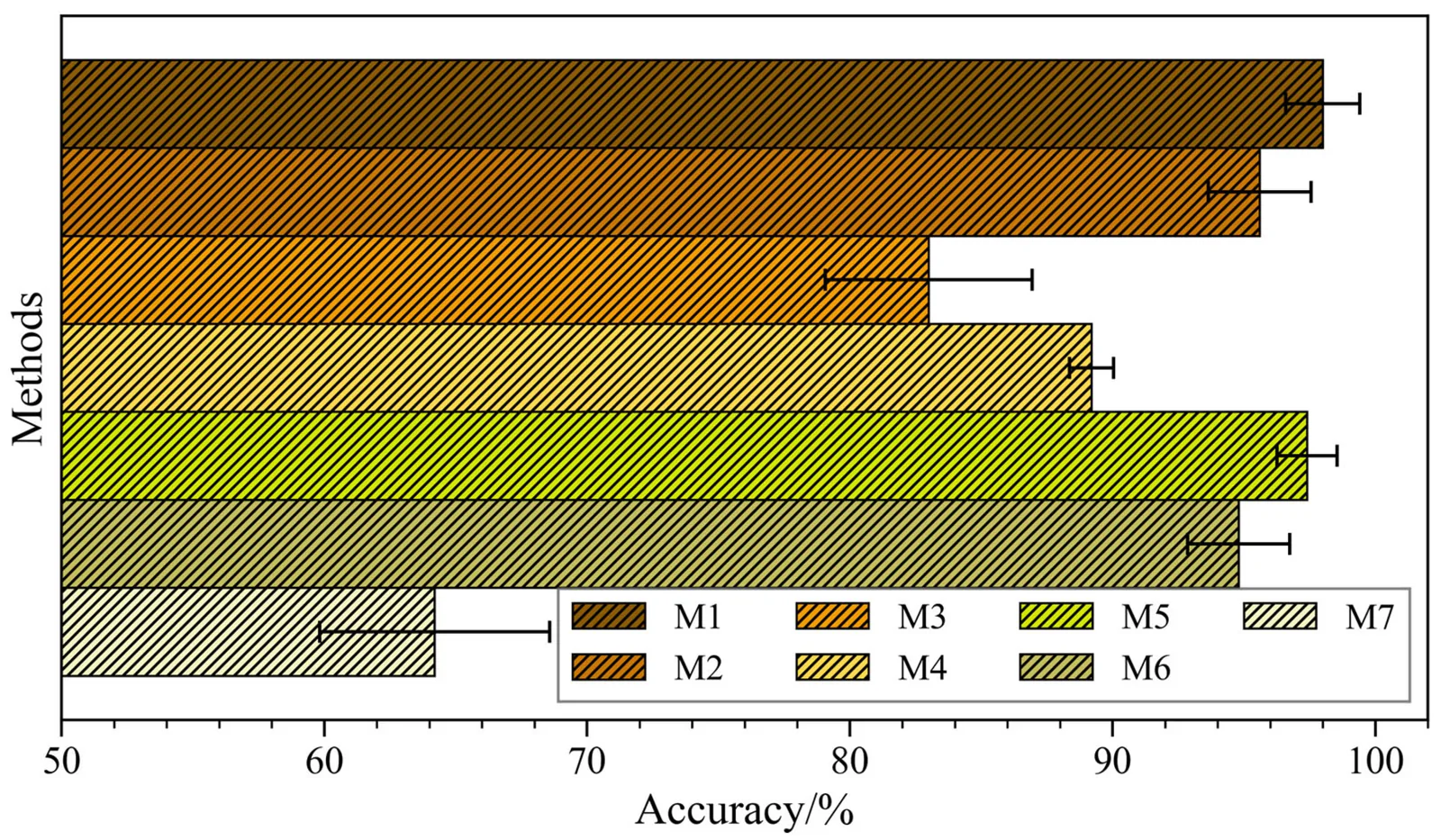
Diagnostic accuracy under different ablation methods.

The ablation results are presented in Table 7 and Figure 12. M1 achieves the highest accuracy of 98.00% ± 1.41%, demonstrating the effectiveness of the complete framework. Compared with M1, M2 obtains 95.60% ± 1.95%, indicating that NARNN-based data augmentation alleviates class imbalance and improves diagnostic performance. Without the SAE, M3 achieves only 83.00% ± 3.94%, confirming the importance of extracting compact and discriminative features before dimensionality reduction by manifold learning. The SAE also reduces the sample dimension from 4000 to 100, substantially decreasing the computational burden of subsequent DTW calculations. M4, which removes DTW-UMAP, obtains 89.20% ± 0.84%, whereas adding conventional UMAP in M5 increases the accuracy to 97.40% ± 1.14%, demonstrating the contribution of manifold learning to neighborhood preservation and feature separability. M1 slightly outperforms M5, suggesting that DTW-based neighborhood construction provides a modest improvement over the Euclidean distance. M6 directly applies DTW-based KNN to the augmented raw signals and achieves a competitive accuracy of 94.80% ± 1.94%, confirming that DTW-based classification is a strong baseline. However, it requires high time consumption and does not produce an explicit low-dimensional representation. M7 obtains the lowest accuracy of 64.20% ± 4.38%, showing that directly applying UMAP to raw signals is ineffective without SAE-based feature extraction. Overall, NARNN alleviates class imbalance, the SAE provides compact feature representations and reduces the cost of DTW calculations, and the combination of DTW-based neighborhood construction and UMAP produces low-dimensional representations with improved class separability.

### 4.2. Case Study 2: Planetary Gearbox Dataset

#### 4.2.1. Dataset Description

**Figure 13:**
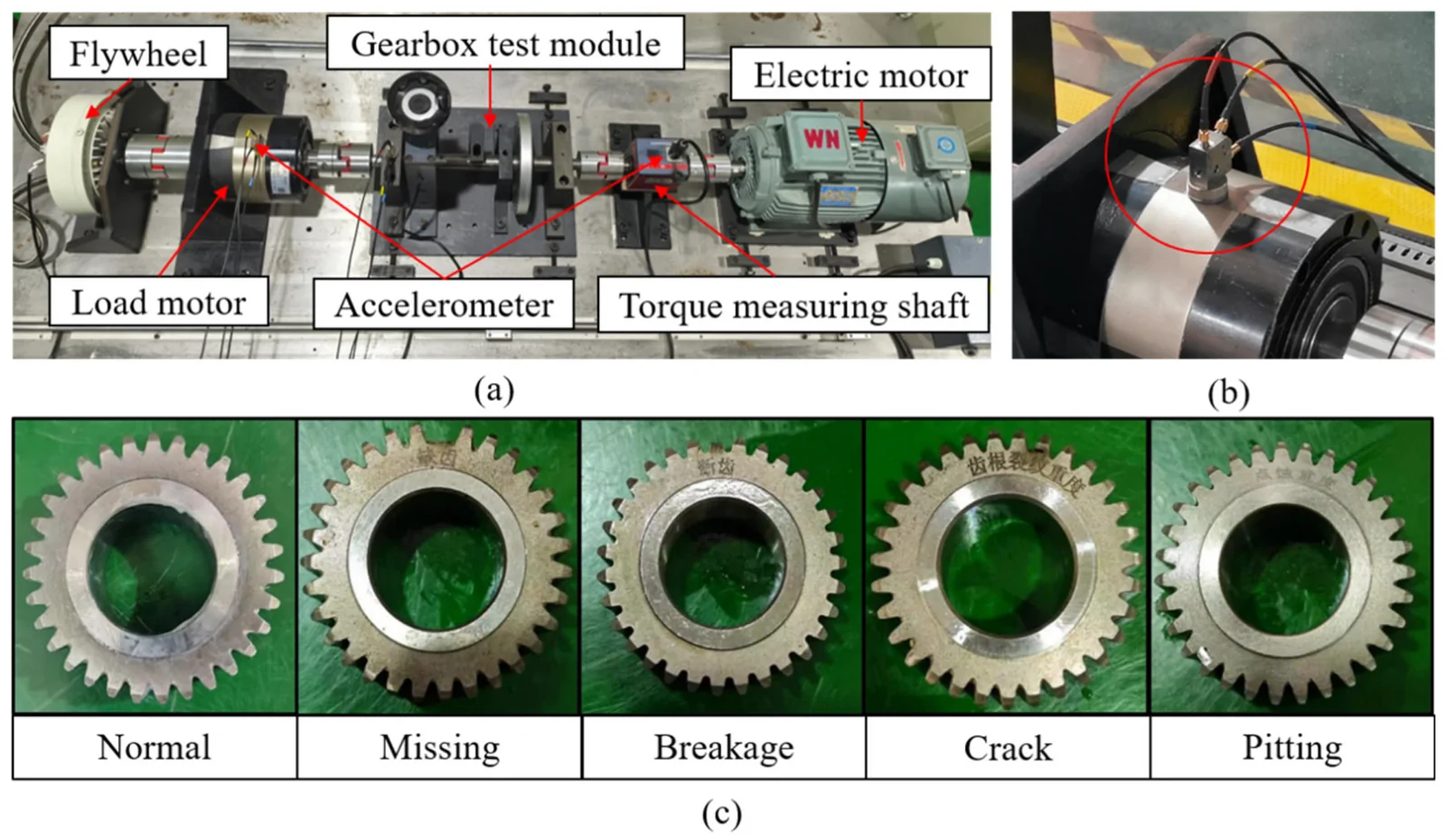
The planetary gearbox test rig: (**a**) configuration of the test rig; (**b**) placement of the accelerometer; (**c**) the health states of gears.

In this case study, the planetary gearbox dataset provided by Wuhan University of Technology was adopted for experimental verification. As illustrated in Figure 13, the test platform mainly included an electric motor, a torque measurement shaft, a gearbox module, a flywheel, and a load motor. Three working conditions were simulated, as summarized in Table 8. During data acquisition, two HDYD232 accelerometers (the Houde test bench, Jiangyin, Jiangsu, China) were mounted on the load motor and torque measurement shaft to record vibration responses in three directions. The sampling frequency was set to 25.6 kHz, and the duration of each test was 2 min. The dataset covered nine health states, including normal, broken tooth, missing tooth, slight tooth-root crack, severe tooth-root crack, slight pitting, severe pitting, slight single-side wear, and severe single-side wear. In this section, the *x*-direction vibration signals of five health states under operating condition 1, including normal, broken tooth, missing tooth, crack and pitting, were selected to construct the experimental dataset. The five health states are depicted in Figure 13c. Each health state contained 3,072,000 data points. The first 327,680 points were used for analysis. After normalization, the signals were segmented by a non-overlapping sliding window of 2048 points, and 160 samples were obtained for each state. Based on these samples, four datasets with imbalance ratios of 1, 0.5, 0.25, and 0.1 were generated, which are denoted as Datasets A, B, C, and D, respectively. After NARNN-based balancing, each minority class in Dataset B contained 80 training samples segmented from the original vibration signals and 80 samples segmented from the NARNN-generated signals. Similarly, each minority class in Dataset C contained 40 original and 120 generated training samples, while each minority class in Dataset D contained 16 original and 144 generated training samples. All testing samples in these datasets were obtained exclusively from the original measured vibration signals. The corresponding numbers and sources of the training and testing samples are described in Table 9.

#### 4.2.2. Performance of the Proposed Method

**Figure 14:**
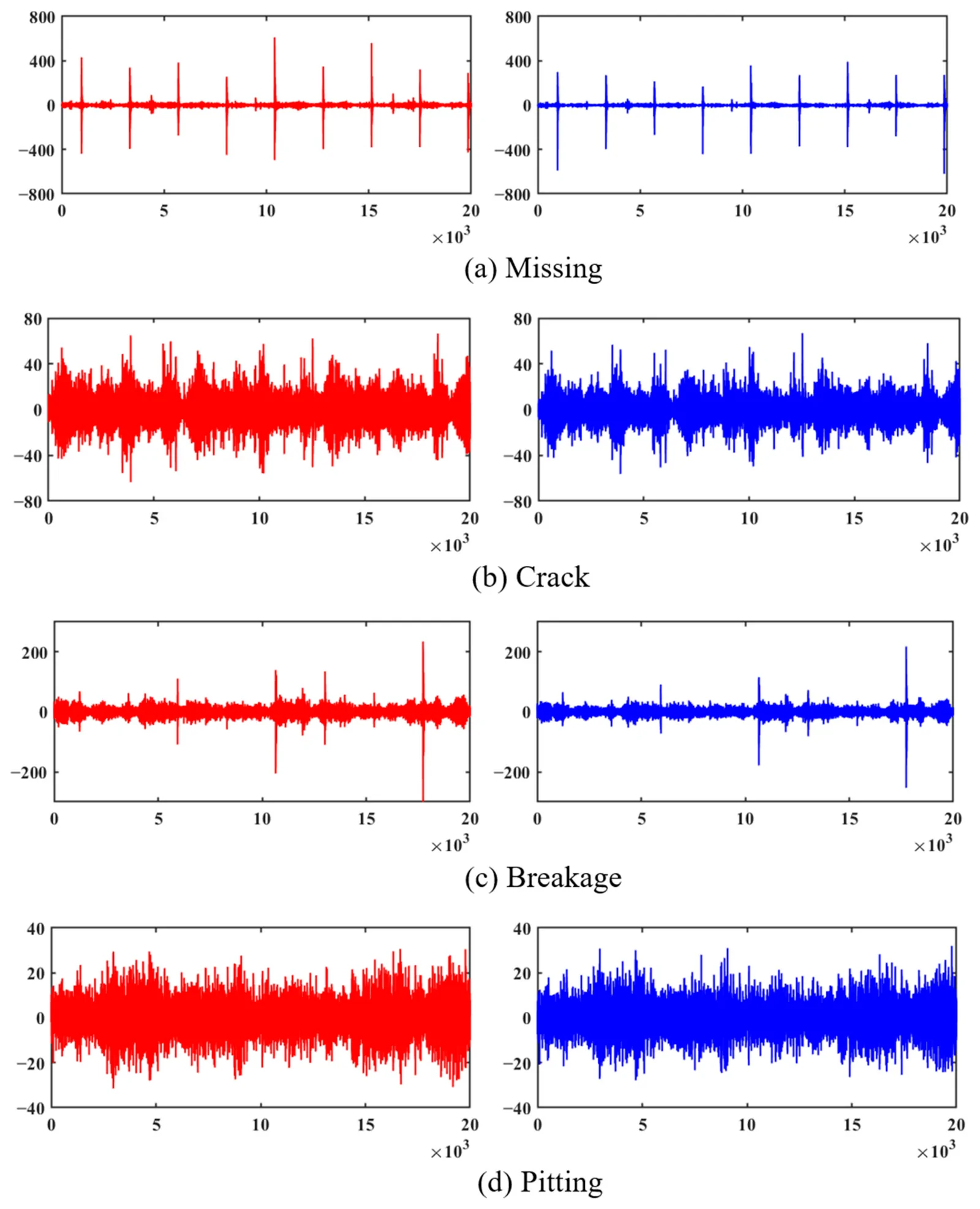
The comparison between original signals and NARNN-generated signals.

**Figure 15:**
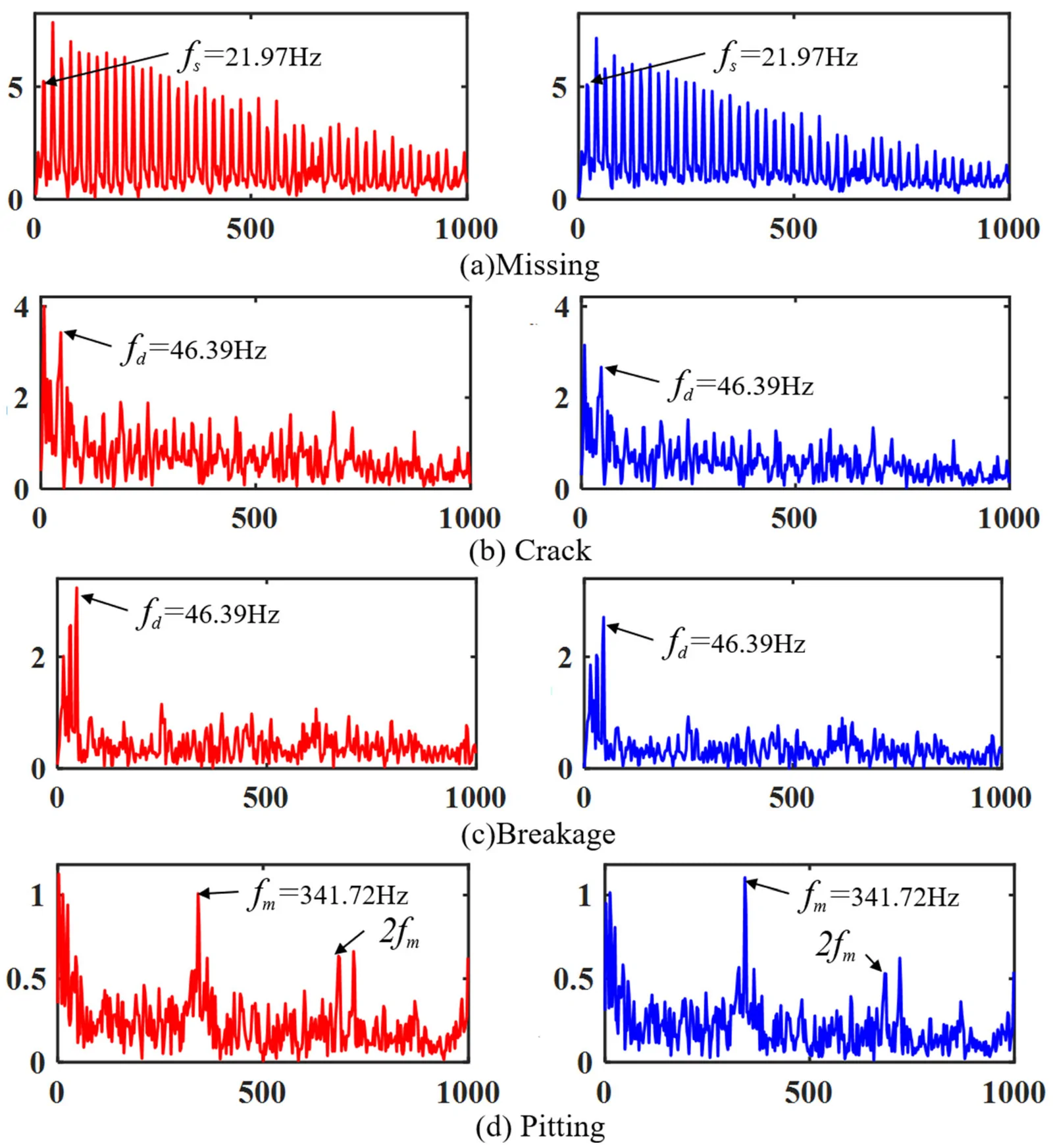
The envelope spectrum comparison between original signals and NARNN-generated signals.

First, the quality of the data generated by the NARNN was evaluated by comparing the generated signals with the corresponding portion of the original measured signal at the same temporal positions from Dataset D, as shown in Figure 14. The generated signals exhibit impact characteristics similar to those of the original signals for different fault types, indicating that the NARNN can effectively capture fault-related features and generate discriminative signals. Furthermore, as shown in Figure 15, the envelope spectra of the generated signals closely agree with those of the original signals, demonstrating the generated signals preserve the main fault-frequency characteristics and spectral distribution of the original vibration signals.

To evaluate the diagnostic effectiveness of the proposed model under imbalanced data conditions, Datasets B, C, and D were processed using the same experimental procedure. The diagnostic results obtained on the different datasets are presented in Table 10. On the balanced Dataset A, a diagnostic accuracy of 99.30% was achieved, demonstrating the strong fault identification capability under balanced class distribution. However, as the degree of imbalance increased, the diagnostic accuracy under imbalanced data gradually decreased. These results indicate that differences in the number of samples among classes reduce interclass separability in the feature space and compromise the stability of the classification results.

After augmentation using the NARNN, the proposed model consistently achieved better diagnostic performance on Datasets B, C, and D than on their imbalanced counterparts. Specifically, the accuracy increased from 97.40% to 98.10% on Dataset B, from 96.00% to 96.60% on Dataset C, and from 93.90% to 95.50% on Dataset D. Similar improvements were observed in Macro-F1 and G-mean. Notably, Dataset D exhibited the largest improvement, with accuracy, Macro-F1, and G-mean increasing by 1.60, 1.59, and 1.69 percentage points, respectively. Meanwhile, the standard deviations of all three metrics decreased substantially for Dataset D, indicating improved diagnostic stability under severe class imbalance. These results demonstrate that NARNN-based augmentation can supplement minority-class samples and alleviate the adverse effects of class imbalance, thereby improving minority-class recognition and overall diagnostic performance.

#### 4.2.3. Comparison with Other Diagnostic Methods

**Figure 16:**
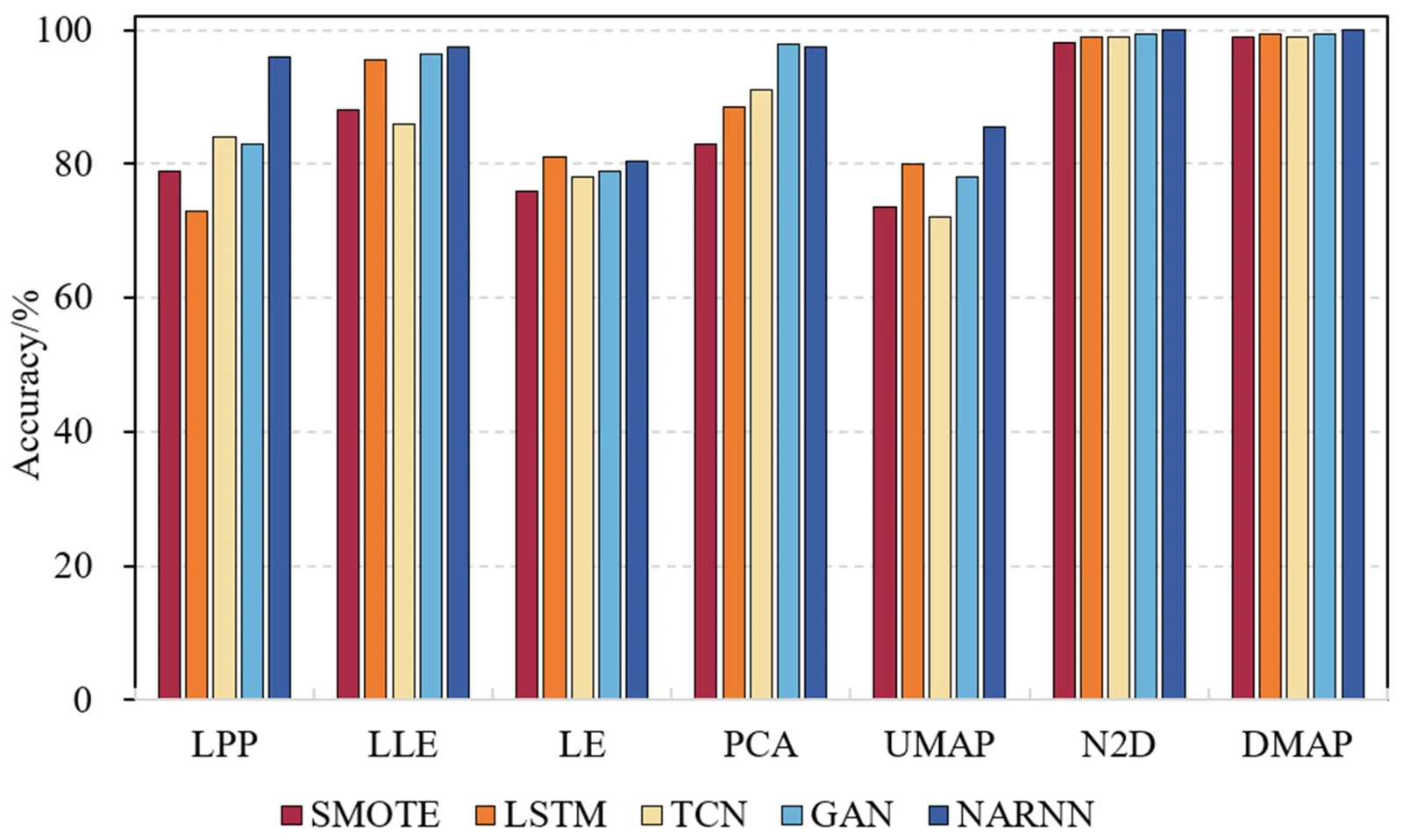
Diagnostic accuracy under different methods.

Comparative experiments were conducted on Dataset D, with the same experimental settings and model parameters used for all methods. Figure 16 presents the diagnostic accuracies obtained by combining different data-generation methods with different dimensionality-reduction methods. The results reveal substantial performance differences among the evaluated combinations. Traditional dimensionality-reduction methods, including LPP, LE, and PCA, exhibit considerable fluctuations in diagnostic accuracy. By contrast, N2D and the proposed DMAP consistently maintain high diagnostic accuracy, demonstrating more stable and effective feature extraction capabilities.

Among the evaluated data-generation methods, the NARNN generally achieves the highest diagnostic accuracy when combined with different dimensionality-reduction methods. This result indicates that its waveform-prediction capability can effectively enrich minority-class samples while retaining fault-related characteristics. Overall, the comparative results demonstrate the effectiveness of the proposed model for fault diagnosis under class-imbalanced conditions.

**Figure 17:**
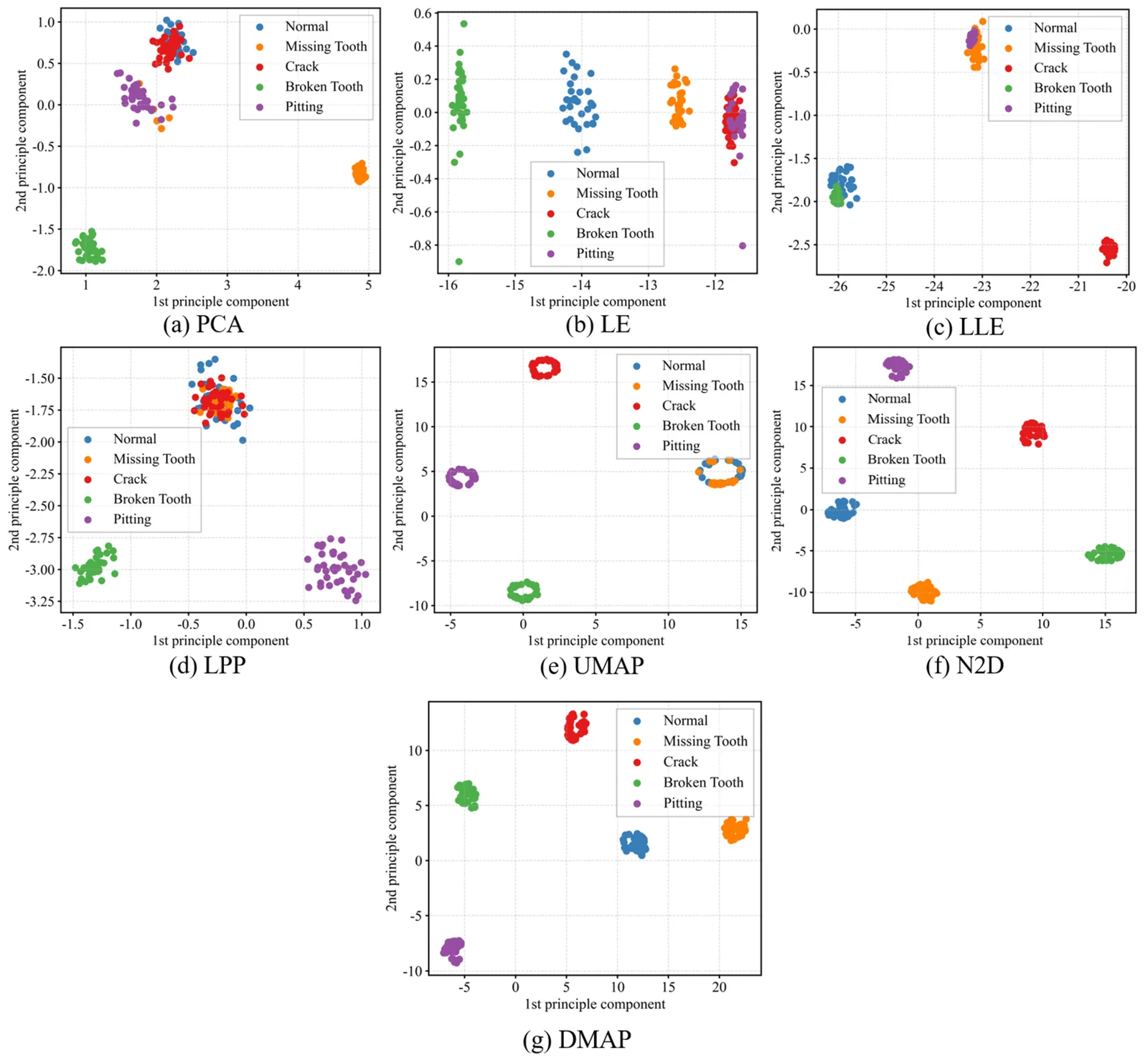
t-SNE visualization of different dimensionality reduction methods on Dataset D.

Figure 17 presents the visualization results obtained using different dimensionality-reduction methods on Dataset D after it was augmented and balanced by the NARNN. As shown in the figure, traditional methods, including LPP, LLE, LE, and PCA, exhibit varying degrees of class overlap in the low-dimensional space. This indicates that linear projection and shallow manifold learning methods have difficulty in fully capturing the nonlinear fault-discriminative information contained in vibration signals. In comparison, UMAP and N2D improve the separability among different fault categories to some extent, producing relatively distinct cluster distributions. The proposed DMAP achieves the best visualization performance, with the five fault categories forming compact and clearly separated clusters in the low-dimensional space. The distinct interclass boundaries indicate that DMAP can effectively preserve the intrinsic manifold structure of fault samples while enhancing the discriminability among different fault categories.

## 5. Conclusions

This study proposes a waveform-prediction augmentation and deep manifold learning framework for imbalanced fault diagnosis in rotating machinery. The framework addresses two major challenges: insufficient representation of minority fault classes and difficulty in extracting discriminative features from high-dimensional vibration signals. First, the NARNN predicts and extends minority-class signals to generate additional training samples and balance the class distribution. The resulting balanced samples are then processed by DMAP for feature extraction and dimensionality reduction. Within DMAP, an SAE extracts compact latent features, DTW calculates pairwise distances for neighborhood construction, and UMAP maps the resulting feature representations into a low-dimensional space. Finally, a KNN classifier identifies the corresponding fault states. Experiments on bearing and gearbox datasets demonstrate that the proposed framework achieves reliable diagnostic performance under class-imbalanced conditions and generally outperforms the comparison methods.

Although the proposed method provides an effective and computationally efficient solution for rotating machinery fault diagnosis under class-imbalanced conditions, its capability under varying operating conditions has not been fully investigated, and the influence of the number and proportion of generated samples on diagnostic performance requires further analysis. Future work will focus on developing more robust strategies that simultaneously consider class imbalance and operating-condition variations, such as cross-condition imbalanced fault diagnosis and domain adaptation techniques. In addition, the effects of different quantities and proportions of generated samples will be systematically evaluated to determine appropriate augmentation strategies and further improve diagnostic reliability under severely imbalanced conditions.

## Figures and Tables

**Table 1:** Detailed description of the bearing working condition in MFPT.

Bearing State	Load /lbs	Abbreviation	Sampling Frequency/sps	Acquisition Time/s	Category Labels
Normal	270	N	97,656	6	0
Outer race fault	100	OR1	48,828	3	1
Outer race fault	200	OR2	48,828	3	2
Inner race fault	300	IR3	48,828	3	3
Inner race fault	0	IR0	48,828	3	4

**Table 2:** Detailed information of the four datasets.

Fault Type	Number of Original/Generated Training Samples				Number of Original/Generated Testing Samples
	Dataset A	Dataset B	Dataset C	Dataset D	Dataset A/B/C/D
N	80/0	80/0	80/0	80/0	20/0
IR0	80/0	60/20	40/40	8/72	20/0
IR3	80/0	60/20	40/40	8/72	20/0
OR2	80/0	40/40	20/60	8/72	20/0
OR1	80/0	40/40	20/60	8/72	20/0

**Table 3:** Overall diagnostic performance under different datasets.

Dataset	Training Data	Accuracy (%)	Macro-F1 (%)	G-Mean (%)
Dataset A	Original balanced	98.20 ± 2.49	98.19 ± 2.52	98.11 ± 2.64
Dataset B	Imbalanced	95.60 ± 1.95	95.55 ± 1.97	95.23 ± 2.17
	NARNN-balanced	98.00 ± 1.41	97.99 ± 1.42	97.90 ± 1.50
Dataset C	Imbalanced	90.60 ± 2.97	90.42 ± 2.92	89.63 ± 3.23
	NARNN-balanced	94.60 ± 3.21	94.47 ± 3.38	94.20 ± 3.61
Dataset D	Imbalanced	67.00 ± 4.64	66.10 ± 4.77	63.14 ± 5.51
	NARNN-balanced	77.20 ± 5.63	76.07 ± 5.58	74.14 ± 5.81

**Table 4:** Parameter settings for the comparative feature extraction methods.

Method	Parameter Settings
PCA	*n*_components_ = 2
LPP	*n*_neighbors_ = 5, *t* = 10, *d*_reduced_ = 2
LLE	*n*_neighbors_ = 5, *d*_reduced_ = 2
LE	*n*_neighbors_ = 9, *d*_reduced_ = 2
UMAP	*n*_neighbors_ = 15, *d*_min_ = 0.1, *d*_reduced_ = 2
N2D	Structure of AE: 512-256-100, *n*_neighbors_ = 15, *d*_min_ = 0.1, *d*_reduced_ = 2
DMAP	Structure of AE: 512-256-100, *n*_neighbors_ = 15, *d*_min_ = 0.1, *d*_reduced_ = 2, metric = DTW distance

**Table 5:** Parameter settings for the comparative data augmentation methods.

Method	Parameter Settings
NARNN	delay order = 10, NHLN = 10
SMOTE	*n*_neighbor_ = 100, *N* = 150%
LSTM	Hidden units = 64, sequence length = 50
TCN	blocks = 4, filters = 64, kernel = 3, dilations = [1, 2, 4, 8], sequence length = 50
GAN	*G*_structure_: 128-256-200, *D*_structure_: 256-128-1, latent dim = 100

**Table 6:** Time consumption of different data augmentation algorithms.

Method	NARNN	SMOTE	LSTM	TCN	GAN
Time consumption/s	3.31	0.019	107.97	25.94	311.62

**Table 7:** Configuration of the ablation methods.

Method	Configuration	Accuracy/%
M1	NARNN + SAE + DTW-UMAP	98.00 ± 1.41
M2	SAE + DTW-UMAP	95.60 ± 1.95
M3	NARNN + DTW-UMAP	83.00 ± 3.94
M4	NARNN + SAE	89.20 ± 0.84
M5	NARNN + SAE + UMAP	97.40 ± 1.14
M6	NARNN + DTW-based KNN	94.80 ± 1.94
M7	NARNN + UMAP	64.20 ± 4.38

**Table 8:** The operating conditions of the planetary gearbox data.

	Condition 1	Condition 2	Condition 3
Rotation speed (rpm)	1200	1500	1800
Torque load (N·m)	7.3	3.6	1

**Table 9:** Detailed information of the imbalanced planetary gearbox data.

Fault Type	Number of Original/Generated Training Samples				Number of Original/Generated Testing Samples
	Dataset A	Dataset B	Dataset C	Dataset D	Dataset A/B/C/D
Normal	160/0	160/0	160/0	160/0	40/0
Missing	160/0	80/80	40/120	16/144	40/0
Crack	160/0	80/80	40/120	16/144	40/0
Breakage	160/0	80/80	40/120	16/144	40/0
Pitting	160/0	80/80	40/120	16/144	40/0

**Table 10:** Diagnostic accuracy under different datasets.

Dataset	Training Data	Accuracy (%)	Macro-F1 (%)	G-Mean (%)
Dataset A	Original balanced	99.30 ± 0.76	99.30 ± 0.76	99.30 ± 0.78
Dataset B	Imbalanced	97.40 ± 1.02	97.39 ± 1.03	97.33 ± 1.05
	NARNN-balanced	98.10 ± 0.65	98.10 ± 0.66	98.06 ± 0.66
Dataset C	Imbalanced	96.00 ± 1.00	95.97 ± 1.02	95.81 ± 1.06
	NARNN-balanced	96.60 ± 1.56	96.56 ± 1.59	96.44 ± 1.69
Dataset D	Imbalanced	93.90 ± 3.60	93.85 ± 3.64	93.60 ± 3.76
	NARNN-balanced	95.50 ± 2.15	95.44 ± 2.17	95.29 ± 2.23

## Data Availability

The data used in Case Study 1 are from the publicly available MFPT bearing dataset, which can be accessed at: https://www.mfpt.org/fault-data-sets/ (accessed on 28 August 2026). The planetary gearbox dataset used in Case Study 2 was obtained from a test rig developed by the authors’ research group. The raw data supporting the conclusions of this article will be made available by the corresponding author upon reasonable request.
